# A Real-World Benchmark of Monte Carlo-Assisted EKF Odometry for Online Pose Estimation in 2D LiDAR SLAM

**DOI:** 10.3390/s26134264

**Published:** 2026-07-04

**Authors:** Andrii Kudriashov, Joanna Koszyk, Bartosz Hyla, Łukasz Ambroziński

**Affiliations:** Department of Robotics and Mechatronics, AGH University of Krakow, al. Mickiewicza 30, 30-059 Kraków, Poland; jkoszyk@agh.edu.pl (J.K.); bhyla@agh.edu.pl (B.H.); ambrozin@agh.edu.pl (Ł.A.)

**Keywords:** SLAM, quadruped robot, mobile robot, localization, multi-sensor fusion, SLAM benchmarking, LiDAR

## Abstract

This study evaluates an Adaptive Monte Carlo Localization-Extended Kalman Filter (AMCL-EKF) pose-estimation stack for repeatable 2D LiDAR SLAM in GPS-denied indoor inspection scenarios. AMCL was used as an online map-referenced correction source fused with LiDAR odometry and Inertial Measurement Unit (IMU) data, and the resulting pose estimate was supplied online to three SLAM backends: Cartographer, GMapping, and SLAM Toolbox. Experiments were performed with a wheeled Husarion Panther and a quadruped Boston Dynamics Spot in three indoor environments of different geometric complexity, producing 720 SLAM executions. Trajectory repeatability was assessed using SE(2)-aligned pairwise and centroid-based ATE-style dispersion and translational RPE, while map repeatability was evaluated with occupied-cell IoU. Accordingly, the metrics were used to quantify between-run dispersion rather than absolute accuracy against external ground-truth data. The results show that AMCL-EKF fusion is highly dependent on the environment, platform, and SLAM backend. AMCL improved selected configurations, especially for Spot in structured environments and for Panther map consistency, but degraded others in geometrically repetitive corridors and mixed-structure spaces. The study also shows that the presence of AMCL-assisted odometry correction alone does not determine final trajectory repeatability, because each SLAM backend incorporates the supplied fused pose estimate differently. The findings support confidence-aware AMCL integration and motivate integrated SLAM architectures resistant to over-correction. These results provide guidance for robust autonomous mapping and inspection with heterogeneous mobile robotic platforms in real environments.

## 1. Introduction

The growing adoption of mobile robotic platforms for environmental mapping, infrastructure inspection, and autonomous exploration has increased the demand for reliable spatial reconstruction and trajectory estimation. Accurate localization and consistent map generation are fundamental for inspection applications, particularly in challenging environments [1,2]. Navigating unknown or geometrically challenging environments also dictates the mechanical design of the robotic platform. Traditional wheeled or tracked robots, while mechanically simple and efficient on flat surfaces [3], frequently fail when confronted with discontinuous terrain, steep stairs, or heavy debris. Legged robots, specifically quadruped platforms, offer a distinct advantage in these domains [4]. By stepping over obstacles rather than rolling over them, legged platforms decouple their trajectory from the immediate roughness of the ground, maintaining stability and mobility where wheeled platforms may lose mobility [5]. The cost of this enhanced mobility is increased mechanical complexity and more dynamic locomotion patterns, which can introduce additional challenges for pose estimation and SLAM [6]. While precise global pose estimation and online path tracking are prerequisites for autonomous operation in the field [7], it remains a major challenge for SLAM in GPS-denied environments, where wheel slip, inertial drift, LiDAR odometry degradation, perceptual aliasing, and geometrically repetitive corridors can lead to inconsistent trajectories and distorted maps. If a robot loses its localization estimate in a dynamic or feature-poor environment, it cannot execute safe recovery or return-to-base procedures [8], accurately map hazards, or complete its designated tasks, ultimately reducing mission reliability and operational safety. Adaptive Monte Carlo Localization [9], or AMCL, traditionally addresses this challenge by providing particle-filter-based localization in a known occupancy-grid map. In the present study, however, AMCL was not supplied with an independently prepared prior map. Instead, in AMCL-enabled runs, it received the occupancy-grid map generated online by the active SLAM backend. Thus, AMCL acted as an online SLAM-map-referenced correction source inside a feedback loop: the SLAM backend incrementally generated the map, AMCL estimated the robot pose with respect to the currently available map, and the resulting pose estimate was delivered to the EKF, from which the fused pose estimate was then supplied back to the SLAM backend as odometry input. The example of that application is the AMCL-EKF odometry stack [10], where, the AMCL, along with laser odometry and IMU data, serves as a correction source for an EKF-based pose estimation to produce a more accurate and globally consistent pose estimate in rough terrain for wheeled robotic platforms.

Recent SLAM research has increasingly moved toward tightly coupled 3D LiDAR-inertial, LiDAR-only, and LiDAR-inertial-visual formulations. Representative examples include factor-graph-based LiDAR-inertial smoothing, iterated-Kalman-filter LiDAR-inertial odometry, lightweight voxel-based LiDAR-inertial odometry, robust ICP-based LiDAR odometry, and tightly coupled LiDAR-inertial-visual mapping systems [11,12,13,14,15,16,17]. These systems are highly relevant for accurate 3D state estimation and large-scale mapping. However, the present work intentionally focuses on a different but still widely deployed class of systems: practical 2D LiDAR occupancy-grid SLAM pipelines used in ROS 1/ROS 2-based indoor navigation and inspection. In such deployments, global map reuse, occupancy-grid repeatability, and stable online pose estimates remain central engineering requirements. Therefore, the aim of this study is not to outperform modern 3D LiDAR-inertial SLAM systems, but to quantify how AMCL-assisted EKF feedback affects the repeatability of representative 2D SLAM backends under real robotic operation.

Performance evaluation of SLAM systems is typically focused on localization accuracy and map quality under single-run experiments [18,19,20,21]. However, in practical robotic deployments, repeatability of trajectory reconstruction across repeated traversals of the same environment is equally important, as it reflects the robustness of the localization pipeline to sensing noise, motion perturbations, and environmental variability [22,23,24,25,26]. To address this, this work introduces a repeatability-oriented evaluation methodology for quantifying trajectory consistency across multiple experimental runs, enabling systematic comparison of SLAM performance under identical operational conditions.

Beyond trajectory repeatability assessment, this work investigates the influence of AMCL-assisted pose correction on trajectory and map consistency within the aforementioned AMCL-EKF approach. While this approach was used in several autonomous wheeled robot applications [27,28], it has not been systematically evaluated from the perspective of run-to-run repeatability, average application-level robustness, application to heterogeneous platforms or multiple SLAM backends, which motivates the present study.

The analysis spans a multi-factor experimental design involving two robotic locomotion modalities, three representative SLAM frameworks, AMCL-enabled and AMCL-disabled localization conditions, repeated traversals in three distinct environments, and multiple stochastic reconstruction runs. This experimental matrix enables systematic quantification of the robustness, variance, and map consistency of each localization configuration under controlled but realistic operating conditions. By comparing representative 2D SLAM pipelines tested on both a quadruped robot (Boston Dynamics Spot) and a wheeled platform (Husarion Panther), we aim to rigorously quantify the effect of continuous map-based feedback. Specifically, we investigate whether AMCL-based global pose correction consistently improves trajectory repeatability and map consistency in challenging real-world conditions, or whether it introduces critical vulnerabilities through map-induced divergence.

Although AMCL, EKF-based sensor fusion, and 2D LiDAR SLAM are well-established techniques, prior studies have typically evaluated these components independently or focused on localization performance within a specific robotic setup. This work instead investigates the consistency of AMCL-assisted EKF pose correction when the same feedback strategy is integrated with multiple SLAM backends and deployed on different robotic platforms. Rather than proposing a new localization or mapping algorithm, the contribution lies in a comparative experimental assessment of how AMCL-EKF feedback influences the reproducibility of trajectory estimates and occupancy-grid maps across repeated runs. This perspective is particularly relevant for inspection robotics, where reliable operation depends not only on accurate localization during a single mission but also on obtaining consistent reconstructions over time.

Because this study focuses on repeatability, internal consistency, and map structural reproducibility across repeated runs, rather than on absolute trajectory or map accuracy against an external reference, ground-truth data were not included. Accordingly, the ATE-style quantities reported in this study quantify SE(2)-aligned dispersion between repeated estimated trajectories; they do not represent error with respect to a true physical trajectory.

The main contributions of this work are threefold:

1. A repeatability-oriented evaluation framework for trajectory consistency assessment;
2. An experimental comparison of wheeled and legged platforms under the same environments, sensing payload, and nominal routes;
3. An analysis of AMCL-assisted localization effects on trajectory and map consistency across multiple 2D SLAM backends and in various environmental conditions.

## 2. Materials and Methods

### 2.1. Site and Equipment Description

**Figure 1:**
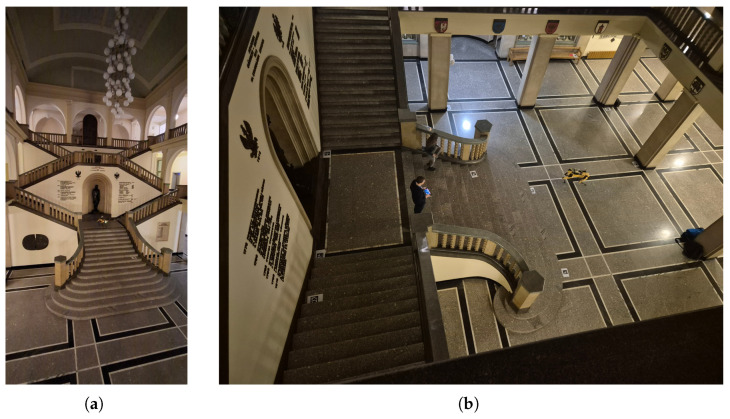
AGH building A0 inspection site (**a**) with Spot and AprilTags visible in (**b**).

**Figure 2:**
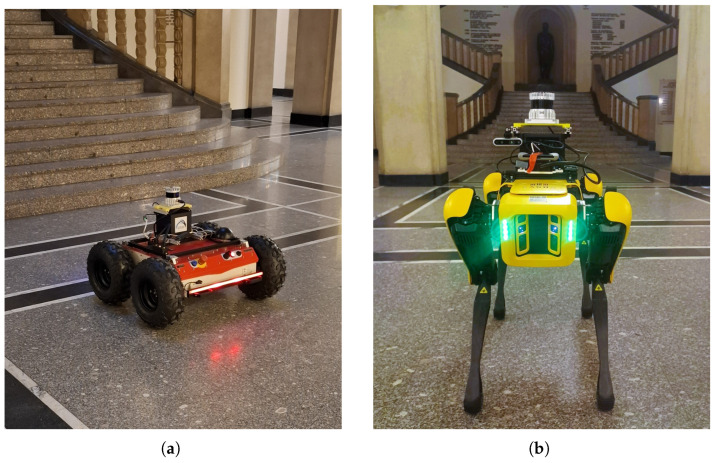
Robotic platforms used in the study: (**a**) wheeled robot Husarion Panther, (**b**) quadruped robot Boston Dynamics Spot.

The experiment was conducted as an indoor inspection scenario in the main A0 building of AGH University of Krakow (Figure 1). The platforms used in the study were wheeled robot Panther from Husarion (Kraków, Poland, Figure 2a) and quadruped robot Spot from Boston Dynamics (Waltham, MA, USA, Figure 2b). Both platforms were equipped with the same replaceable payload: an Ouster OS-1 3D LiDAR (Ouster, Inc., San Francisco, CA, USA) with 64 channels and a built-in 6-DOF IMU connected to a Jetson NVIDIA embedded computing unit (Nvidia Corp., Santa Clara, CA, USA). The system is based on Ubuntu 24.04 and ROS 2 (Jazzy), and the devices are time-synchronized via a hardware clock to ensure consistent LiDAR/IMU fusion.

To ensure measurement repeatability and reduce human interference, we defined a mission in Spot’s native software version 5.1.0. by Boston Dynamics (Waltham, MA, USA). First, we placed AprilTag markers in the environment, which was necessary to perform a mission. Markers were used by the robot as reference points for recording and repeating the mission. Then, the robot’s path was defined by manually operating the robot and recording the path. After that, the waypoints determining the robot’s behavior during mission replay were saved. In the next stage, the robot was able to perform fully autonomous operation without human supervision and repeat the entire route twice while recording raw sensor data. Panther’s navigation was human-assisted on the same nominal route.

**Figure A12:**
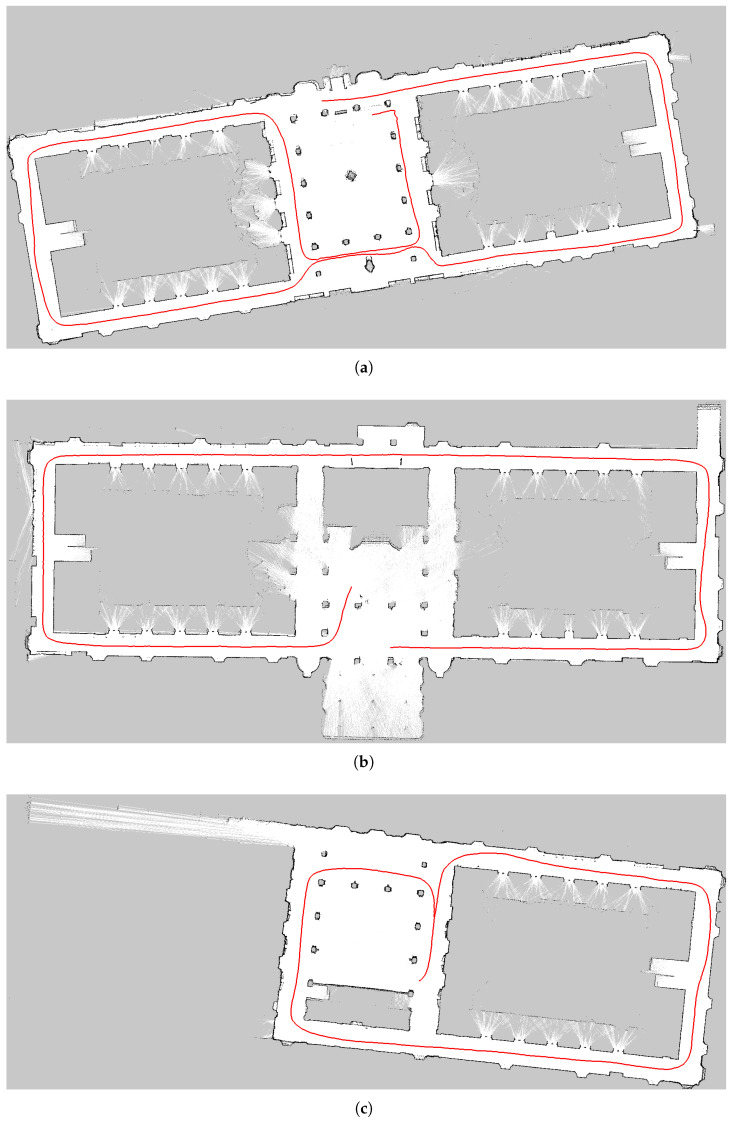
Maps generated by SLAM Toolbox under AMCL-enabled conditions (single run): (**a**) A-1, second floor; (**b**) B-1, ground floor; (**c**) C-1, first floor. The red line corresponds to the robot’s path, estimated during the run.

The collected measurements were performed in three experimental environments located on different floors of the A0 building, denoted as A, B, and C (see Figure A12 in Appendix A for representative maps of each).

- Environment A is a geometrically challenging, long-corridor section characterized by repetitive, featureless walls and limited structural landmarks. It features the longest traversal paths, making it the most demanding scenario for scan-matching and particle-filter convergence due to high susceptibility to geometric aliasing.
- Environment B is a topologically stable, feature-rich section of the building that includes offices, open-plan areas, and visually distinct architectural elements. It features a high density of reliable LiDAR landmarks and provides potentially favorable conditions for SLAM-based global localization.
- Environment C is an intermediate, mixed-structure space combining corridor and room segments across moderate path lengths. It lacks both the extreme geometric ambiguity of Environment A and the landmark richness of Environment B, resulting in varied and algorithm-dependent AMCL behavior.

We performed two independent measurements per environment per platform, resulting in 12 (3 environments × 2 measurements × 2 platforms) distinct raw datasets. Each measurement was processed through a test pipeline that executed a full SLAM session for three algorithms, with 10 iterations per algorithm under AMCL-enabled and AMCL-disabled conditions. This resulted in 2×30 runs for each of 12 datasets, or 720 SLAM executions, including repeated stochastic processing runs for each raw dataset (Table 1). To isolate the core algorithmic performance from potential hardware-level inefficiencies (e.g., suboptimal memory allocation or poor resource management typical of embedded systems), a separate high-performance workstation was utilized for all computations. The hardware configuration consisted of an AMD Ryzen 7 5800X processor, 32 GB of DDR4 RAM, a Radeon RX 6700 XT graphics card with 12 GB of GDDR6 memory, and a 1 TB NVMe SSD. The software environment was built on Arch Linux, utilizing Docker containers to host the ROS 2 framework.

### 2.2. 2D SLAM Stack

**Figure 3:**
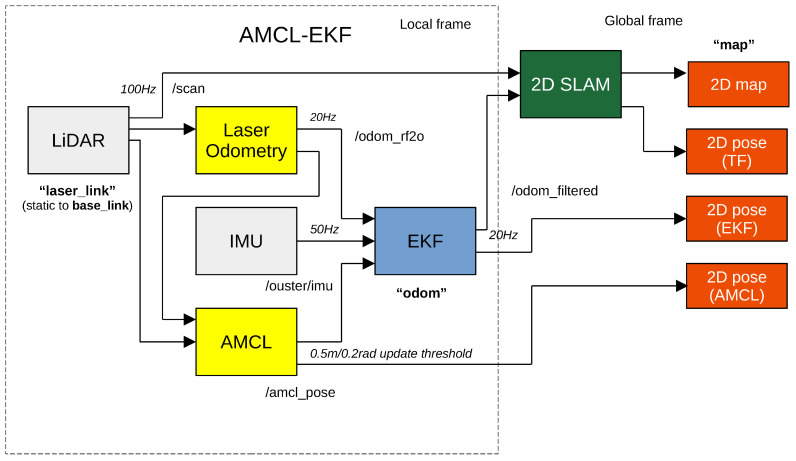
AMCL-EKF pose-estimation pipeline. LiDAR odometry, IMU data, and AMCL pose corrections are fused in the EKF. The fused pose estimate is supplied to the SLAM backend, which generates the evaluated trajectory and occupancy-grid map. The main coordinate frames are map (global frame), odom (local frame), and base_link (robot and payload frame).

The AMCL-EKF pose estimation pipeline (Figure 3) fuses IMU-LiDAR-based odometry with AMCL pose corrections, providing a continuous global pose estimate to the SLAM backend [10]. The EKF serves as a state estimator that integrates high-frequency odometry measurements with lower-frequency, map-based pose corrections from AMCL. Following our original work [10], the EKF implementation by Moore et al. [29] was applied, while the RF2O algorithm [30] was used for planar laser odometry. The motivation for this selection can be found in [10,31]. This fusion allows for improved trajectory stability and drift correction, particularly in feature-rich environments where AMCL can provide reliable global pose updates. The resulting pose estimates are then utilized by the SLAM backend to construct occupancy grid maps and optimize the robot’s trajectory over time.

In this paper, the terms *AMCL-enabled* and *AMCL* refer to the same SLAM execution condition, while *AMCL-disabled* and *no-AMCL* refer to the corresponding condition without AMCL feedback. The shorter labels *AMCL* and *no-AMCL* are used primarily in equations, tables, figures, and plot legends to improve readability and accommodate limited display space.

#### 2.2.1. SLAM Algorithm Selection

For this evaluation, we surveyed the most widely adopted 2D SLAM algorithms with open-source availability in the ROS 2 ecosystem. To ensure comparability across implementations, we enforced three additional selection criteria: support for external odometry input, occupancy grid map generation, and native 2D LiDAR scan processing. This led to the decision to evaluate the following algorithms:

- Cartographer—an algorithm developed by Google, commonly used over the past 10 years [32] (ROS 1 with an official release for ROS 2, plus an additional extension for 3D mapping and improvements [33]).
- GMapping—the most well-known and widely used ROS 1 SLAM algorithm that produces occupancy grid 2D maps [34,35] (no official ROS 2 support, based on an open-source fork [36]).
- SLAM Toolbox—a robust 2D SLAM algorithm that became a widely used SLAM framework and reference in modern ROS 2-based mobile robotics solutions (ROS 2 support only) [37].

#### 2.2.2. Configuration and Tuning Strategy

The same localization and SLAM parameter sets were used for all repeated executions within a given backend in order to isolate the effect of AMCL-EKF correction from run-specific manual tuning. No environment-specific optimization was performed after inspecting the results. The AMCL configuration used a fixed particle-filter setup, likelihood-field laser model, and fixed update thresholds, while the EKF fused RF2O laser odometry, IMU measurements, and, in AMCL-enabled runs, AMCL pose corrections. Backend-specific parameters for Cartographer, GMapping, and SLAM Toolbox were kept constant across AMCL-enabled and AMCL-disabled modes. For comparability, all three SLAM backends were configured with a map resolution of 0.05 m and equivalent motion-update thresholds of 0.5 m in translation and 0.2 rad in rotation. In GMapping, these correspond to *linearUpdate* and *angularUpdate*; in SLAM Toolbox, to *minimum_travel_distance* and *minimum_travel_heading*; and in Cartographer, to the 2D trajectory-builder motion-filter parameters *max_distance_meters* and *max_angle_radians*. Although the internal use of these parameters may differ between backends, they provide a consistent basis for scan insertion/update triggering across the evaluated pipelines. The main parameters are summarized in Table 2.

### 2.3. Repeatability Evaluation of Pose Estimation

#### 2.3.1. Relation to Modern SLAM Benchmarking

Recent SLAM benchmarking practice increasingly emphasizes three complementary aspects: (i) standardized trajectory evaluation with explicit alignment assumptions and error definitions [20,38]; (ii) robustness-aware reporting (including incomplete or failed runs), not only nominal accuracy [39,40,41]; and (iii) repeated-route assessment, where *precision* (repeatability) is often more relevant than one-shot absolute accuracy for dependable field deployment [42,43]. In line with this state of the art, our methodology centers on repeatability across repeated traversals, while retaining completeness, statistical uncertainty, and effect-size reporting for practical interpretation.

#### 2.3.2. Data and Preprocessing

For each SLAM backend a∈A and localization condition c∈{AMCL,no−AMCL}, we collect $K_{a,c}$ repeated traversals of the same route. Each run is represented as a planar trajectory

(1) $$P^{\left(k\right)}={\left\{p_{i}^{\left(k\right)}\right\}}_{i=1}^{N},\;p_{i}^{\left(k\right)}\in R^{2},$$

after resampling to a common length *N* via interpolation over a shared progress variable (*time* or *arc length*) to ensure pointwise correspondence across runs. Arc-length resampling is used when speed profiles differ (our case), improving robustness of pointwise comparisons [38]. This resampling step is critical for enabling direct pointwise comparison of trajectories, as it accounts for differences in speed profiles and ensures that corresponding points represent the same relative position along the route across all runs.

The resulting aligned trajectories are then used for subsequent repeatability analysis. Repeatability is defined as dispersion across repeated runs within the same (a,c) condition.

#### 2.3.3. Alignment and Trajectory Metrics

Because scale is observable in metric 2D LiDAR/IMU SLAM, trajectories are aligned in SE(2) (rotation + translation, no scaling). For two trajectories $A={\left\{a_{i}\right\}}_{i=1}^{N}$ and $B={\left\{b_{i}\right\}}_{i=1}^{N}$, we estimate $\left(\hat{R},\hat{t}\right)$ by

(2) $$\left(\hat{R},\hat{t}\right)=\arg\min_{R\in SO\left(2\right),\;t\in R^{2}}\sum_{i=1}^{N}{∥b_{i}-\left(Ra_{i}+t\right)∥}^{2}.$$

The solution is computed with SVD and fixed unit scale [38,44]. The primary trajectory metric is ATE (RMSE):

(3) $$e_{i}=b_{i}-\left(\hat{R}a_{i}+\hat{t}\right),$$

(4) $${\mathrm{ATE}}_{\mathrm{RMSE}}\left(A,B\right)=\sqrt{\frac{1}{N}\sum_{i=1}^{N}{∥e_{i}∥}^{2}}.$$

As a local-consistency complement, we report a translational RPE-style proxy over interval Δ:

(5) $${\mathrm{rpe}}_{i}=∥\left(p_{i+\Delta }-p_{i}\right)-\left(q_{i+\Delta }-q_{i}\right)∥,$$

(6) $${\mathrm{RPE}}_{t,\mathrm{RMSE}}=\sqrt{\frac{1}{N-\Delta }\sum_{i=1}^{N-\Delta }{\mathrm{rpe}}_{i}^{2}}.$$

#### 2.3.4. Repeatability Statistics Within Each Condition

##### Pairwise Run-to-Run Repeatability

For each unordered pair (k,ℓ), k<ℓ, within condition (a,c), we compute pairwise ATE:

(7) $$d_{k\ell }^{\left(a,c\right)}={\mathrm{ATE}}_{\mathrm{RMSE}}\;\left(P^{\left(k\right)},P^{\left(\ell \right)}\right).$$

This defines

(8) $$\begin{array}{c} D_{a,c}=\left\{d_{k\ell }^{\left(a,c\right)}|1\leq k<\ell \leq K_{a,c}\right\},\\ \;\left|D_{a,c}\right|=\frac{K_{a,c}\left(K_{a,c}-1\right)}{2}. \end{array}$$

Lower values indicate higher repeatability.

##### Centroid Repeatability and Completeness

To obtain approximately independent run-level values for inference, we also compute each run’s deviation from a mean trajectory ${\bar{P}}_{a,c}$:

(9) $$g_{k}^{\left(a,c\right)}={\mathrm{ATE}}_{\mathrm{RMSE}}\;\left(P^{\left(k\right)},{\bar{P}}_{a,c}\right),$$

and report run completeness

(10) $$C^{\left(k\right)}=\frac{N_{\mathrm{valid}}^{\left(k\right)}}{N_{\mathrm{ref}}},$$

where $N_{\mathrm{ref}}$ is the median valid length for the route. We additionally report success rate, i.e., the fraction of runs with $C^{\left(k\right)}\geq C_{\min}$.

#### 2.3.5. Comparing AMCL and No-AMCL Conditions: Uncertainty, Tests, and Effect Size

We compare $D_{a,\mathrm{AMCL}}$ and $D_{a,\mathrm{no}\;-\;\mathrm{AMCL}}$ using 95% bootstrap confidence intervals for the mean (percentile method, *B* resamples) [45]. Pairwise ATE values are not statistically independent because individual runs contribute to multiple comparisons; therefore, they are interpreted primarily as descriptive measures of trajectory dispersion. AMCL-vs-no-AMCL comparisons reported for pairwise ATE, including Welch’s *t*-test [46] and Cliff’s δ, are therefore treated as descriptive distributional comparisons. To provide an additional run-level comparison, formal hypothesis testing is performed on the run-level centroid deviations $\left\{g_{k}^{\left(a,c\right)}\right\}$ using the Mann–Whitney *U* test [47], with inferential conclusions based on run-level summaries whenever possible. Practical significance is quantified with Cliff’s δ, a non-parametric dominance effect-size measure that complements rank-based group comparisons and summarizes the degree of distributional non-overlap [48]. Similar reporting has been used for sensor-derived motion and signal-quality metrics [49,50]:

(11) $$\begin{array}{c} \delta =\frac{\#(x>y)-\#(x<y)}{mn},\\ m=|D_{a,\mathrm{AMCL}}\left|,\;n=\right|D_{a,\mathrm{no}\;-\;\mathrm{AMCL}}|. \end{array}$$

Here δ<0 indicates lower dispersion under AMCL-enabled conditions. When centroid-based effect sizes are reported, the same dominance statistic is applied to the centroid-deviation samples instead of the pairwise ATE samples.

### 2.4. Repeatability Evaluation of 2D Occupancy Maps

#### 2.4.1. Map Representation and Preprocessing

Each run outputs a ROS occupancy grid map. Cell probabilities p(u,v)∈[0,1] are discretized using thresholds $t_{\mathrm{occ}}$ and $t_{\mathrm{free}}$ into occupied, free, or unknown [51]:

(12) $$m\left(u,v\right)=\left\{\begin{array}{cc} \mathrm{occ}, & p\left(u,v\right)\geq t_{\mathrm{occ}}\\ \mathrm{free}, & p\left(u,v\right)\leq t_{\mathrm{free}}\\ \mathrm{unk}, & \mathrm{otherwise}. \end{array}\right.$$

Before comparison, maps are aligned in SE(2) using ICP on occupied/boundary point sets to remove global translation/rotation offsets [52].

All similarity metrics are computed only on cells known in both maps:

(13) $$\Omega =\{\left(u,v\right)|m_{A}\left(u,v\right)\neq \mathrm{unk}\;\wedge \;m_{B}\left(u,v\right)\neq \mathrm{unk}\}.$$

#### 2.4.2. Map Similarity Metrics

The primary map-repeatability metric is occupied-cell IoU, also known as the Jaccard coefficient [53,54,55]:

(14) $${\mathrm{IoU}}_{\mathrm{occ}}\left(A,B\right)=\frac{|A_{\mathrm{occ}}\cap B_{\mathrm{occ}}|}{|A_{\mathrm{occ}}\cup B_{\mathrm{occ}}|},$$

defined over Ω.

#### 2.4.3. Repeatability Statistics

For each algorithm *a* and condition *c*, with maps ${\left\{M^{\left(k\right)}\right\}}_{k=1}^{K_{a,c}}$, we compute

(15) $$S_{a,c}=\left\{{\mathrm{IoU}}_{\mathrm{occ}}\;\left(M^{\left(k\right)},M^{\left(\ell \right)}\right)\;|\;1\leq k<\ell \leq K_{a,c}\right\}.$$

As with trajectory comparisons, pairwise IoU values share common maps and are therefore interpreted primarily as descriptive measures of map-structure repeatability; inferential conclusions are treated cautiously and supported by run-level or condition-level summaries where possible. We report means with 95% bootstrap confidence intervals and AMCL-vs-no-AMCL comparisons using Welch’s test, Mann–Whitney *U*, and Cliff’s δ [45,46,47,48,49,50].

### 2.5. Velocity Profiling

Velocity profiling was included as a supplementary evaluation because the two platforms did not execute the route under identical control conditions. Spot followed a replayed autonomous mission, whereas Panther was human-assisted along the same nominal path. Prior work shows that tracking accuracy depends on translational velocity and trajectory geometry, and that locomotion-induced body motion can degrade LiDAR-SLAM map quality. More broadly, SLAM performance is affected by platform motion through point-cloud distortion, turning behavior, and aggressive motion [56]. For that reason, differences in translational speed, yaw rate, stopping behavior, and local trajectory shape may explain part of the observed SLAM variability independently of the SLAM algorithms themselves.

Linear velocity was not estimated directly from raw 6-DOF IMU measurements. Although accelerometer data can in principle be integrated to obtain velocity and position, inertial-only estimates are highly sensitive to sensor noise, bias, attitude error, and imperfect gravity compensation, and the resulting drift grows rapidly over time. This limitation is especially problematic for mobile robots executing long runs and for legged platforms whose gait introduces repeated impacts, vibration, and body-axis oscillations [57]. Accordingly, IMU-only velocity was considered unsuitable as the primary basis for comparing repeated navigation trials. Instead, velocity estimates were derived from timestamped pose trajectories available in TF. This provides a common pose-based representation for all platforms, environments, algorithms, AMCL configurations, and repeated trials, while avoiding unbounded inertial integration drift [58]. Linear velocity was computed from successive pose displacements over time, and angular velocity was computed from successive changes in yaw. This choice should be interpreted as a consistent internal kinematic proxy rather than as a direct physical measurement of platform motion.

These TF-derived velocities are not ground truth. They inherit the limitations of the underlying localization or SLAM estimate, including pose noise, nonuniform update timing, and corrections introduced by relocalization, loop closure, or graph optimization. Previous works show that global correction stages can run asynchronously and may modify previously estimated poses or introduce sudden jumps in the trajectory [59]. Therefore, the reported velocity profiles are used here as comparative descriptors of executed motion as observed in the pose estimate, not as absolute reference measurements. When a continuous local odometry or fused odometry stream is available, it is preferable as the primary source for velocity profiling; otherwise, TF-based profiles remain useful provided this limitation is stated explicitly.

## 3. Experimental Results

In accordance with the proposed methodology, we evaluated the trajectory repeatability of three distinct 2D SLAM frameworks utilizing both a wheeled platform (Husarion Panther) and a quadruped robot (Boston Dynamics Spot) across three environments. Our primary objective is to quantify SE(2)-aligned trajectory dispersion between repeated estimates, rather than absolute trajectory error with respect to ground truth, and to determine whether continuous map-based AMCL feedback improves online pose-estimation repeatability across different locomotion modalities.

### 3.1. IMU Measurements Impact on Trajectory Repeatability and Map Consistency

To quantify motion-induced disturbance as a potential source of SLAM repeatability differences, we analyzed the raw IMU streams from Environment A, Measurement 1 for both platforms. For each record, we computed sampling characteristics and summary statistics of angular-rate and linear-acceleration signals directly from the raw time series (Table 3).

Both logs were sampled at approximately 50 Hz, but with different traversal durations (Panther: 206.37 s, Spot: 316.50 s). The Spot platform exhibited substantially stronger translational excitation: the mean lateral acceleration magnitude $\sqrt{a_{x}^{2}+a_{y}^{2}}$ increased from 2.80 to 4.27 m/s^2^ (+52.3%), and its 95th percentile increased from 5.92 to 10.20 m/s^2^ (+72.3%). Likewise, the deviation of acceleration norm from gravity, $\left|∥a∥-9.81\right|$, increased from 1.31 to 2.29 m/s^2^ in the mean (+74.8%) and from 3.43 to 8.75 m/s^2^ at the 95th percentile (+154.9%). These values indicate markedly higher body excitation for the legged platform, consistent with step-induced oscillations.

**Figure 4:**
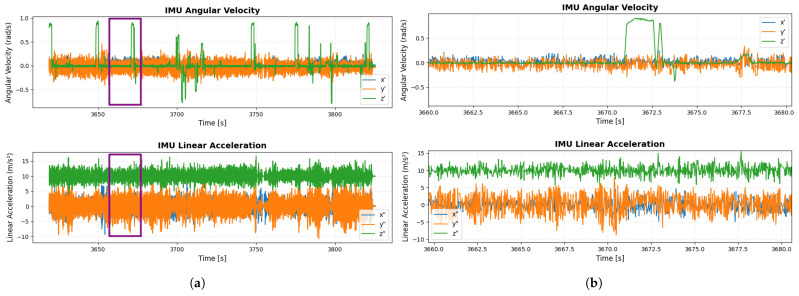
Measured IMU acceleration and angular velocity for Panther during Environment A, Measurement 1: (**a**) full trajectory with selected segments highlighted for analysis, (**b**) zoomed segments for detailed inspection.

**Figure 5:**
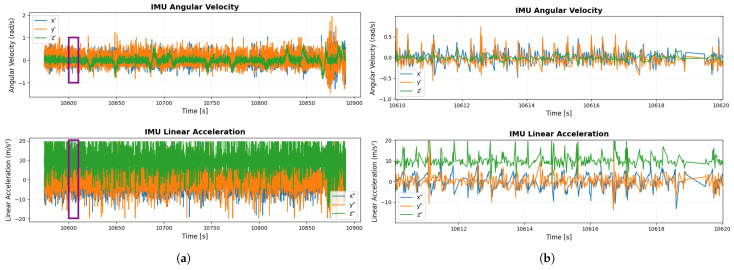
Measured IMU acceleration and angular velocity for Spot during Environment A, Measurement 1: (**a**) full trajectory with selected segments highlighted for analysis, (**b**) zoomed segments for detailed inspection.

Rotational dynamics showed a mixed pattern (Figure 4 and Figure 5). Spot had a higher mean angular-speed norm (0.261 vs. 0.170 rad/s, +52.9%), but lower 95th-percentile angular speed (0.621 vs. 0.827 rad/s, −24.9%), while exhibiting larger isolated peaks (2.22 vs. 0.95 rad/s). This suggests that Panther experiences more persistent yaw-rich motion in the upper quantiles during turning, whereas Spot is characterized by intermittent high-intensity rotational spikes associated with gait transitions and local terrain interactions.

From a localization perspective, the IMU evidence supports the interpretation used in subsequent trajectory/map analyses: (i) larger high-frequency translational excitation in Spot can smear projected 2D scan alignment and increase map inconsistency in repetitive corridors; (ii) Panther’s lower translational excitation but sustained yaw activity may favor stable scan registration in structured corridors while remaining sensitive to long-horizon wheel-slip accumulation. Therefore, AMCL impact should be interpreted jointly with platform-specific inertial disturbance profiles rather than as a purely algorithmic effect.

### 3.2. Operator Influence Evaluation via Velocity Profiles

The collected results were divided into Platform-Algorithm-Mode-Environment-Measurement groups, for which both linear and angular velocity profiles were calculated from the TF trajectories generated during each SLAM execution. Since each run provides two profiles (linear and angular), the complete analysis contains 720×2=1440 velocity profiles. To preserve readability, we include only a representative example for one environment–measurement–algorithm pair in the Appendix A, while the complete set of profiles is provided in the published dataset.

**Figure 6:**
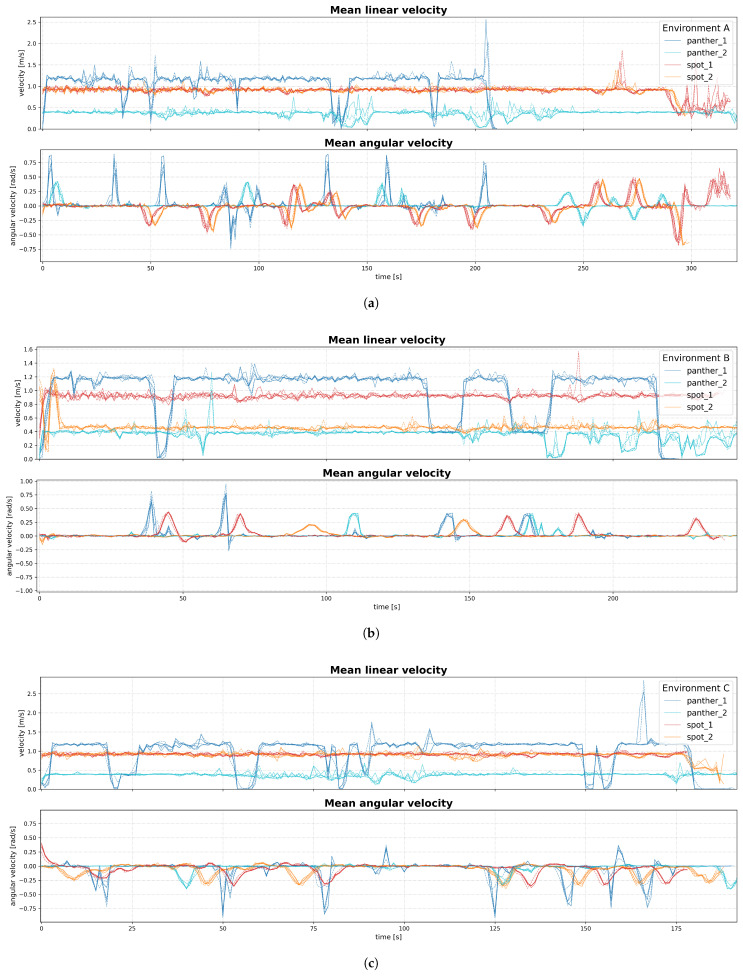
Mean linear and angular velocities calculated for each measurement/platform pair per environment: (**a**) Environment A, (**b**) Environment B, (**c**) Environment C. Solid lines correspond to AMCL-enabled mode, dashed to AMCL-disabled mode.

Calculated mean profiles over time for each environment are shown in Figure 6, and the corresponding mean values with standard deviations are summarized in Table 4. The results show highly consistent Spot velocity profiles between Measurements 1 and 2 in Environments A and C, which is expected for a predefined route executed autonomously. In contrast, Panther shows a clear difference between Measurements 1 and 2 in the same environments: Measurement 1 is faster, whereas Measurement 2 is slower and longer. This behavior is consistent with human-assisted driving and operator-dependent speed selection. Although the nominal path remained unchanged, these velocity profiles introduced additional execution variability within the same environment, thereby broadening the evaluation conditions. Environment B represents a different pattern. Measurement 1 is relatively fast for both platforms (about 1.0–1.2 m/s), while Measurement 2 is slower for both Panther and Spot (about 0.3–0.5 m/s), which reflects the experimental execution conditions. This combination strengthens the analysis by ensuring that comparable speed modes are represented across both platforms under identical environmental conditions. The angular velocity statistics further support this pattern. Panther generally shows lower angular velocities in the slower Measurement 2 runs, especially in Environments A, B, and C, which is consistent with slower and more cautious manual operation. Spot maintains more consistent angular velocity levels between Measurements 1 and 2 in Environments A and C, reflecting the repeatability of autonomous mission execution. These differences are important because angular velocity affects scan overlap, local geometric observability, and the difficulty of scan matching during turns. All observations were verified against the trajectory-scale statistics to confirm that the observed trends were not artifacts of the velocity calculation. Overall, the velocity-profile analysis shows that platform operation mode is a relevant factor in the interpretation of SLAM repeatability results. Part of the observed variation should therefore be attributed not only to the SLAM algorithm or platform morphology, but also to the temporal and kinematic characteristics of how each trajectory was executed.

### 3.3. Path Length Consistency and Traversal Scale

To isolate algorithmic performance from pure odometric drift, it is necessary to contextualize the repeatability errors within the physical scale of the experiments. Table 5 presents the unified path length statistics for both platforms.

The environments represented significantly diverse traversals. Environment A required extensive mean path lengths ranging from approximately 216 m to 277 m, Environment C spanned 163 m to 181 m, and Environment B covered longer traversals for both platforms, from approximately 219 m up to 287 m. Notably, the standard deviations within each measurement subset are extremely tight for both platforms (ranging from 1.94 m to 8.11 m).

Because the total path lengths within each measurement subset were relatively consistent, large trajectory discrepancies are unlikely to be caused solely by gross traversal-scale differences. However, path length alone does not guarantee identical motion execution; local curvature, stopping behavior, angular velocity, and operator-induced deviations may still influence scan matching and SLAM repeatability.

### 3.4. Trajectory Overlap Evaluation

**Figure 7:**
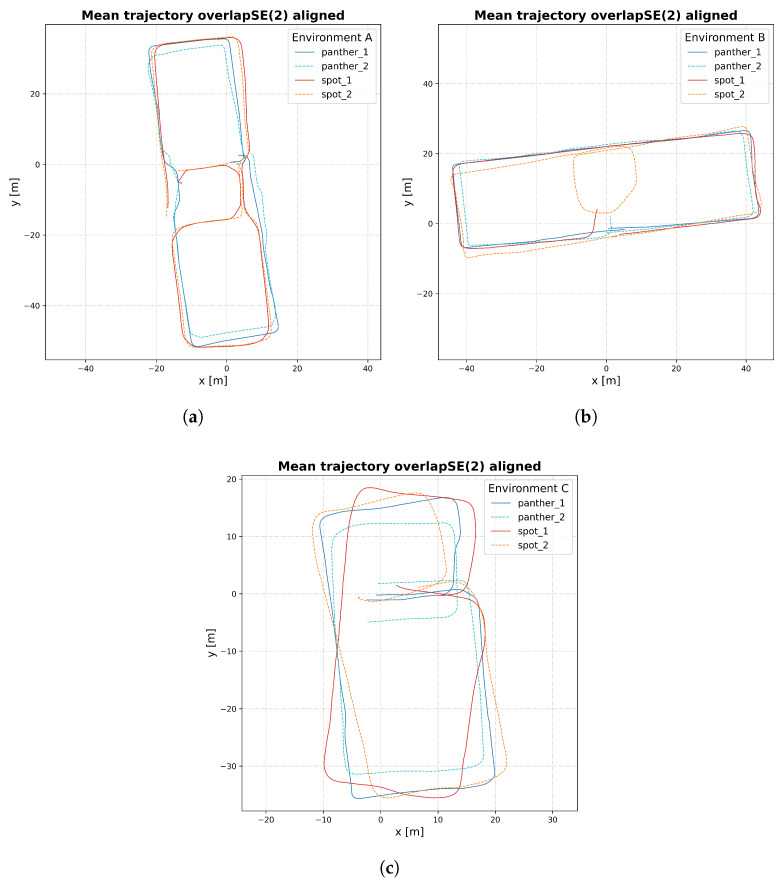
Mean trajectory overlaps aligned in SE(2) per environment for all measurements: (**a**) Environment A, (**b**) Environment B, (**c**) Environment C.

The trajectory-overlap analysis complements the scalar path-length and velocity-profile summaries by showing how the mean route geometry changes between platforms and measurement repetitions. For this purpose, the mean trajectories for each platform–measurement pair were aligned in SE(2) before visualization. This alignment removes the arbitrary global translation and heading offset between maps and emphasizes residual differences in route shape, local curvature, loop closure, and traversal scale. Therefore, the plots in Figure 7 should not be interpreted as absolute global localization accuracy, but as a visual measure of how consistently the same physical route was reconstructed by the SLAM executions.

It is important to distinguish this visualization from the pairwise spatial-deviation statistics calculated inside each platform–measurement–algorithm–mode condition. The latter quantify run-to-run repeatability under the same experimental condition, whereas Figure 7 compares the mean trajectories between different platform–measurement pairs. Consequently, a condition can be internally repeatable while still producing a mean route that differs from the other platform or measurement.

Environment B provides the most consistent cross-platform and cross-measurement overlap. The four mean trajectories follow the same elongated outer loop after SE(2) alignment, and the main visible discrepancy is the additional central excursion in Spot Measurement 2. This excursion is consistent with the larger path length observed for this measurement, but it is systematic rather than random: the repeated executions within the same condition remain spatially stable. Thus, Environment B provides the cleanest visual comparison of platform effects, because most of the route geometry is shared by all platform–measurement pairs.

Environment A shows an intermediate behavior. The general corridor-loop structure is preserved, and the two Spot measurements overlap closely with each other, while the Panther measurements also remain mutually similar. However, platform-dependent offsets appear along the long straight segments and around the central transition. This means that Environment A contains both a stable nominal route shape and local geometry-dependent disagreements. The pairwise spatial-deviation statistics still identify Spot Measurement 1 as a high-dispersion case within repeated SLAM executions, but the mean-overlap plot does not indicate the same level of cross-platform shape inconsistency as observed in Environment C.

Environment C is the least consistent environment in the mean-overlap visualization. Although all trajectories correspond to the same nominal route, the aligned curves differ noticeably around the upper loop, central crossing, and lower return segment. Unlike Environment A, the discrepancy is not concentrated in one platform–measurement pair; instead, all four mean trajectories contribute to the spread. A supplementary nearest-trajectory check on the aligned mean curves supports this visual interpretation, giving the largest average shape discrepancy for Environment C (approximately 2.08 m), compared with Environment A (approximately 1.64 m) and Environment B (approximately 1.21 m).

Overall, the overlap analysis shows that Environment B has the highest cross-platform route-shape consistency, Environment A is mostly consistent but affected by platform-dependent local offsets and high within-condition dispersion for Spot Measurement 1, and Environment C exhibits the weakest agreement between platform–measurement mean trajectories. Therefore, the later ATE-based results for Environment C should be interpreted with particular attention to route-shape differences introduced by execution mode, turning geometry, and local SLAM reconstruction, rather than only as differences in algorithmic repeatability.

### 3.5. Quantitative Evaluation of Cross-Platform Trajectory Repeatability

A comprehensive summary of the aggregated repeatability metrics for both platforms is presented in Table 6. The combined data reveals a critical insight: the efficacy of AMCL is dictated by a complex interaction between the spatial structure of the environment and the underlying kinematic stability of the robot.

**Figure 8:**
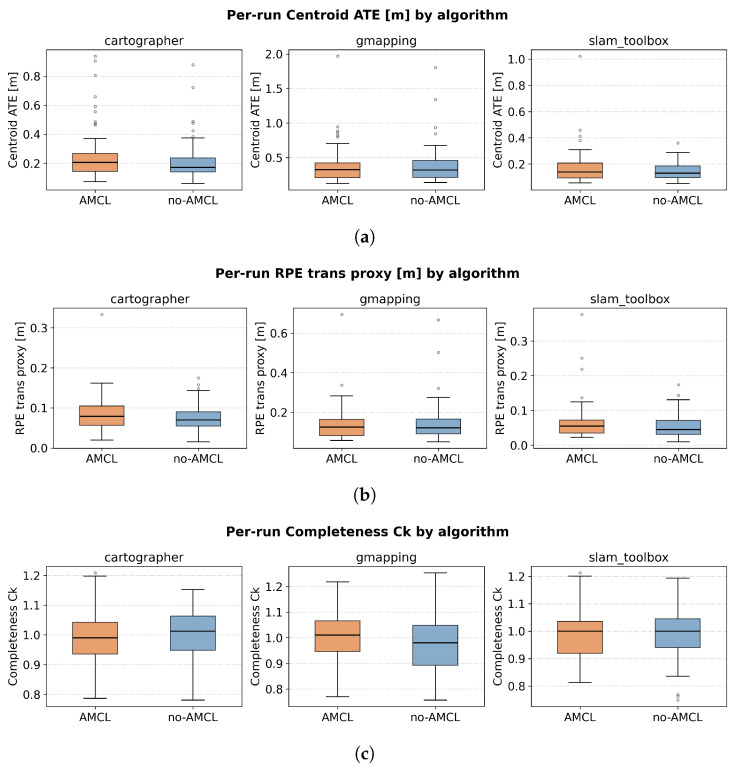
Run-level trajectory statistics for Panther, aggregated over all environment-measurement subsets for each SLAM backend and localization condition: (**a**) centroid ATE-style dispersion relative to the condition mean trajectory, (**b**) translational RPE-style local-consistency error, and (**c**) trajectory-completeness ratio. Boxes compare AMCL-enabled and AMCL-disabled runs. Lower values in (**a**,**b**), and higher values in (**c**), indicate more repeatable or complete trajectory reconstruction.

**Figure 9:**
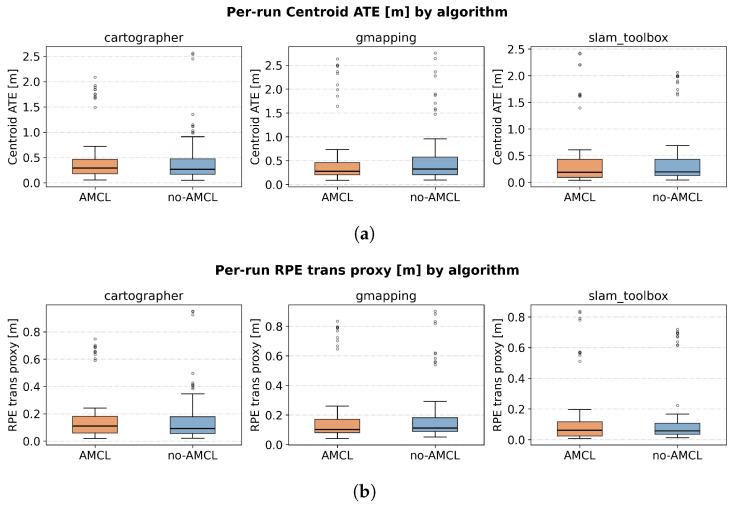

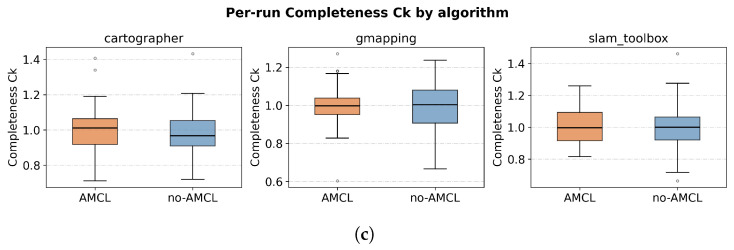
Run-level trajectory statistics for Spot, aggregated over all environment–measurement subsets for each SLAM backend and localization condition: (**a**) centroid ATE-style dispersion relative to the condition mean trajectory, (**b**) translational RPE-style local-consistency error, and (**c**) trajectory-completeness ratio. Boxes compare AMCL-enabled and AMCL-disabled runs. Lower values in (**a**,**b**), and higher values in (**c**), indicate more repeatable or complete trajectory reconstruction.

Figure 8 and Figure 9 decompose the aggregate statistics of Table 6 into individual run distributions, revealing the within-condition variability that mean values alone conceal. For the Panther (Figure 8), the centroid ATE boxplots confirm that most runs remain in the sub-meter range across the tested environments, with stronger variability appearing in selected algorithm-environment combinations. The pairwise ATE summary in Table 6 shows that AMCL does not act as a uniform error reducer for the wheeled platform. In Environment A1, AMCL increases Cartographer dispersion from 0.294 m to 0.662 m, while reducing GMapping dispersion from 0.962 m to 0.809 m. In Environment C2, AMCL substantially increases dispersion for GMapping and SLAM Toolbox. The RPE panel indicates that local step-to-step tracking remains comparatively stable across most runs, while the ATE statistics reveal that global trajectory repeatability is strongly dependent on the SLAM backend and environment. Completeness scores are uniformly near 1.0, indicating that path truncation does not bias the error statistics.

For the Spot (Figure 9), the per-run ATE distributions are systematically higher in Environment A (medians roughly 1.5–2.5 m across algorithms), consistent with the aggregate figures in Table 6, and the interquartile ranges are comparably wide under AMCL-enabled and AMCL-disabled conditions. This contrasts with the Panther, where AMCL visibly tightened the spread. In Environments B and C, Spot’s per-run ATE drops to sub-0.5 m, and the AMCL/no-AMCL box overlap is large, reflecting the modest and mixed effect sizes reported in Table 6. The RPE distributions for Spot are notably broader than those of the Panther in long-corridor environments, consistent with the higher inertial excitation quantified in Table 3, and completeness exhibits occasional sub-unity values primarily in geometrically aliased segments of Environment A.

**Figure 10:**
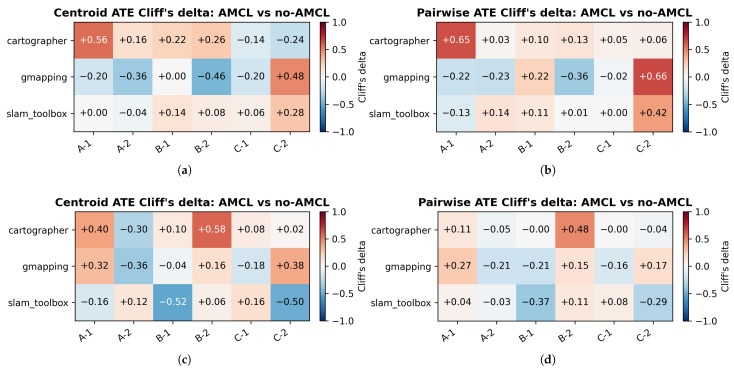
Cliff’s δ heatmaps for SE(2)-aligned trajectory-repeatability metrics. Panther: (**a**) run-level centroid ATE-style dispersion, (**b**) pairwise run-to-run ATE-style dispersion; Spot: (**c**) run-level centroid ATE-style dispersion, (**d**) pairwise run-to-run ATE-style dispersion. Negative values indicate lower trajectory dispersion in the AMCL-enabled condition, whereas positive values indicate lower dispersion in the no-AMCL condition.

To facilitate a rapid cross-environment and cross-algorithm overview, Figure 10 presents the Cliff’s δ effect sizes from Table 6 as colour-coded heatmaps. Each cell encodes the direction and magnitude of AMCL’s influence on ATE for a given environment–measurement–algorithm combination: negative (blue) cells indicate a reduction in trajectory error, while positive (red) cells indicate degradation. The centroid and pairwise variants of the metric are shown side by side to distinguish systematic bias from run-to-run dispersion effects.

#### 3.5.1. Topologically Stable Environments (Environment B)

In Environment B, AMCL’s effect on trajectory repeatability is platform-dependent, while its effect on map consistency is more uniformly positive. For the wheeled Panther in Measurement 1, GMapping’s pairwise ATE slightly increased from 0.573 m to 0.636 m (Cliff’s δ=+0.225). In contrast, Spot showed a clear improvement, with GMapping’s error falling from 0.525 m to 0.361 m (δ=−0.213). This platform divergence suggests that map-based correction benefits the legged platform more in this structured environment, while the wheeled platform’s GMapping trajectory variability was not reduced by AMCL correction.

#### 3.5.2. High-Drift and Long-Corridor Environments (Environment A)

Environment A highlights the strongest influence of geometrically repetitive corridors on AMCL-assisted localization. For the wheeled Panther in Measurement 1, AMCL increases Cartographer pairwise ATE from 0.294 m to 0.662 m, indicating a clear degradation in trajectory repeatability. In the same measurement, however, GMapping decreases from 0.962 m to 0.809 m, while SLAM Toolbox increases from 0.198 m to 0.330 m. This shows that the AMCL effect is not purely platform-dependent but also strongly coupled to the SLAM backend.

In Measurement 2, Panther Cartographer remains nearly unchanged under AMCL, with pairwise ATE changing only from 0.326 m to 0.333 m. GMapping improves from 0.543 m to 0.460 m, whereas SLAM Toolbox degrades from 0.230 m to 0.282 m. Therefore, for the wheeled platform, AMCL should be interpreted as a conditional correction source rather than a universal drift-recovery mechanism.

For the legged Spot, Environment A exhibits a more systematic degradation pattern in Measurement 1. AMCL increases pairwise ATE for all three algorithms: Cartographer from 1.867 m to 2.196 m, GMapping from 2.448 m to 2.794 m, and SLAM Toolbox from 2.250 m to 2.260 m. This suggests that, in long and geometrically ambiguous corridors, continuous map-based corrections can amplify trajectory variability on the legged platform, most likely due to particle-filter ambiguity and scan-to-map over-correction.

Overall, Environment A demonstrates that AMCL-EKF fusion must be applied selectively. It can reduce dispersion for selected backend/platform combinations, but it can also increase trajectory variability when the map-based correction is affected by corridor aliasing or platform-induced motion disturbances.

#### 3.5.3. Mixed-Structure Medium-Length Environments (Environment C)

Environment C represents an intermediate case: a mixed-structure indoor space with moderate path lengths (≈163–182 m, Table 5) that lacks both the featureless long corridors of Environment A and the high landmark density of Environment B. As a result, AMCL’s influence on trajectory repeatability is more equivocal, with modest and often statistically non-significant effect sizes across most configurations (Table 6).

For the wheeled Panther, the picture is split by algorithm. Cartographer shows only a negligible AMCL effect in both measurements (pairwise ATE 0.421 m vs. 0.419 m, δ=+0.052 in Meas. 1; 0.381 m to 0.338 m, δ=+0.061 in Meas. 2). GMapping is near-neutral in Measurement 1 (0.472→0.462 m, δ=−0.024) but exhibits a severe degradation in Measurement 2 (0.441→0.812 m, δ=+0.662). SLAM Toolbox likewise shows near-zero sensitivity in Measurement 1 (δ=+0.001) but a notable degradation in Measurement 2 (0.217 m to 0.333 m, δ=+0.418), suggesting that in this mixed-structure environment AMCL can introduce additional variability in the wheeled platform rather than suppress drift.

For the legged Spot, the results are broadly consistent with the Panther in Environment C. Cartographer again shows near-zero sensitivity to AMCL in both measurements (δ=−0.000 and −0.036), reflecting its robust submap-based backend. GMapping exhibits a slight improvement in Measurement 1 (0.455→0.402 m, δ=−0.160) but a marginal degradation in Measurement 2 (δ=+0.168). SLAM Toolbox benefits from AMCL in Measurement 2 (0.281 → 0.220 m, δ=−0.294), in contrast to the Panther result, where the same backend degrades from 0.217 m to 0.333 m. This indicates that the AMCL effect in mixed-structure environments is not purely algorithm-specific, but depends on the interaction between platform dynamics, environment structure, and SLAM backend behavior. Across Environment C, the results therefore represent an intermediate but still heterogeneous case: some configurations remain nearly unchanged, while others show substantial degradation or improvement under AMCL.

### 3.6. Map Structural Consistency (Occupied IoU)

To complement the trajectory analysis, we evaluated the structural repeatability of the generated spatial models by calculating the pairwise Intersection over Union (IoU) of the occupied map cells. The IoU metric quantifies how consistently an algorithm places physical obstacles across multiple independent runs. A higher mean IoU indicates that the generated map is structurally stable, whereas a low IoU implies geometric blurring, map rotation, or inconsistent obstacle plotting.

A comprehensive summary of the pairwise occupied IoU metrics for both the Husarion Panther (wheeled) and the Boston Dynamics Spot (legged) is presented in Table 7.

**Figure 11:**
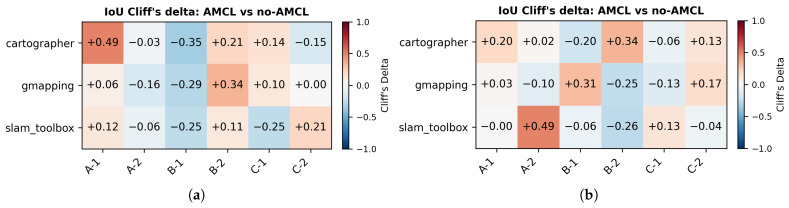
Combined Map Cliff’s δ heatmap: (**a**) Husarion Panther, (**b**) Boston Dynamics Spot. Negative values indicate that AMCL improves map consistency, while positive values indicate degradation.

An at-a-glance summary of AMCL’s structural impact is provided by Figure 11, which encodes the Cliff’s δ effect sizes from Table 7 as colour-coded heatmaps for both platforms. As with the trajectory heatmaps, negative (blue) cells indicate that enabling AMCL improves map consistency (higher IoU), while positive (red) cells indicate structural degradation. Placing the Panther and Spot panels side by side makes the environment- and platform-specific patterns immediately visible, highlighting where the two locomotion modalities diverge in their response to map-based pose correction.

**Figure 12:**
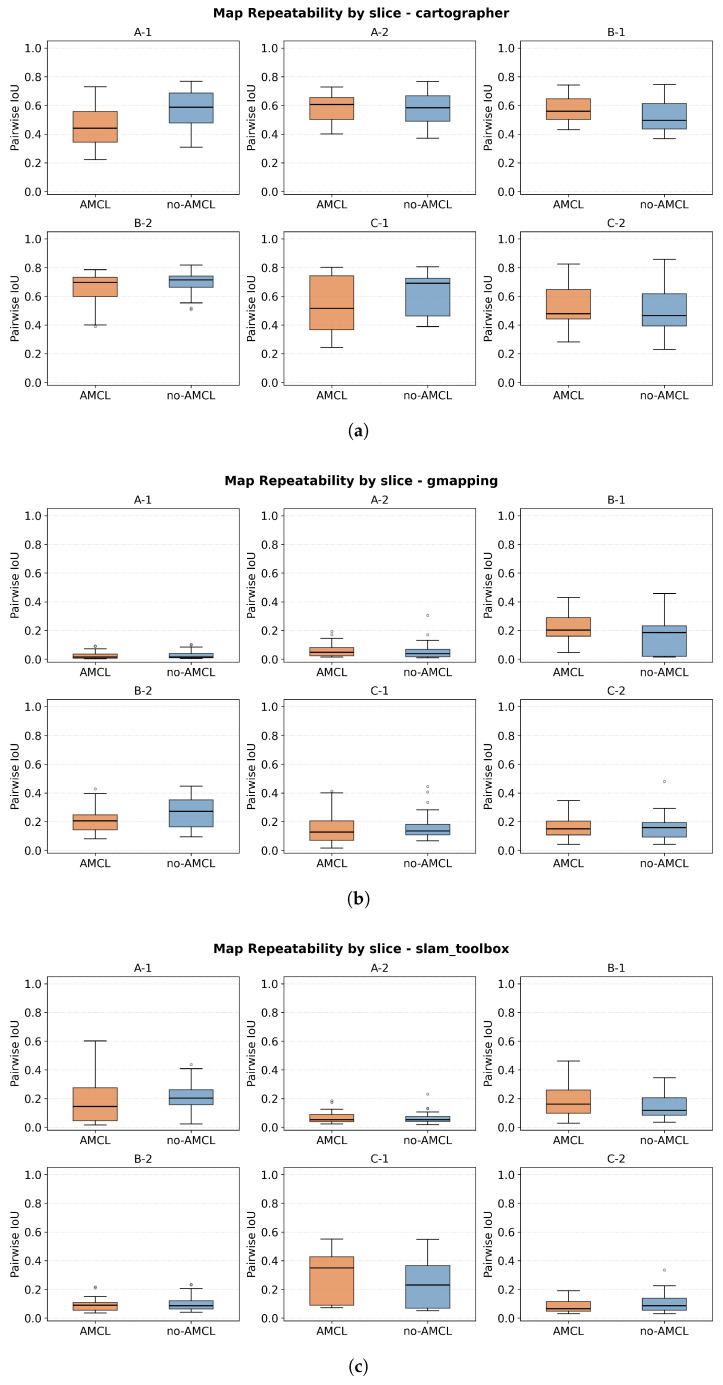
AMCL influence on Mean IoU by measurement slice across environments for wheeled Panther and different SLAM backends: (**a**) Cartographer, (**b**) GMapping, (**c**) SLAM Toolbox.

**Figure 13:**
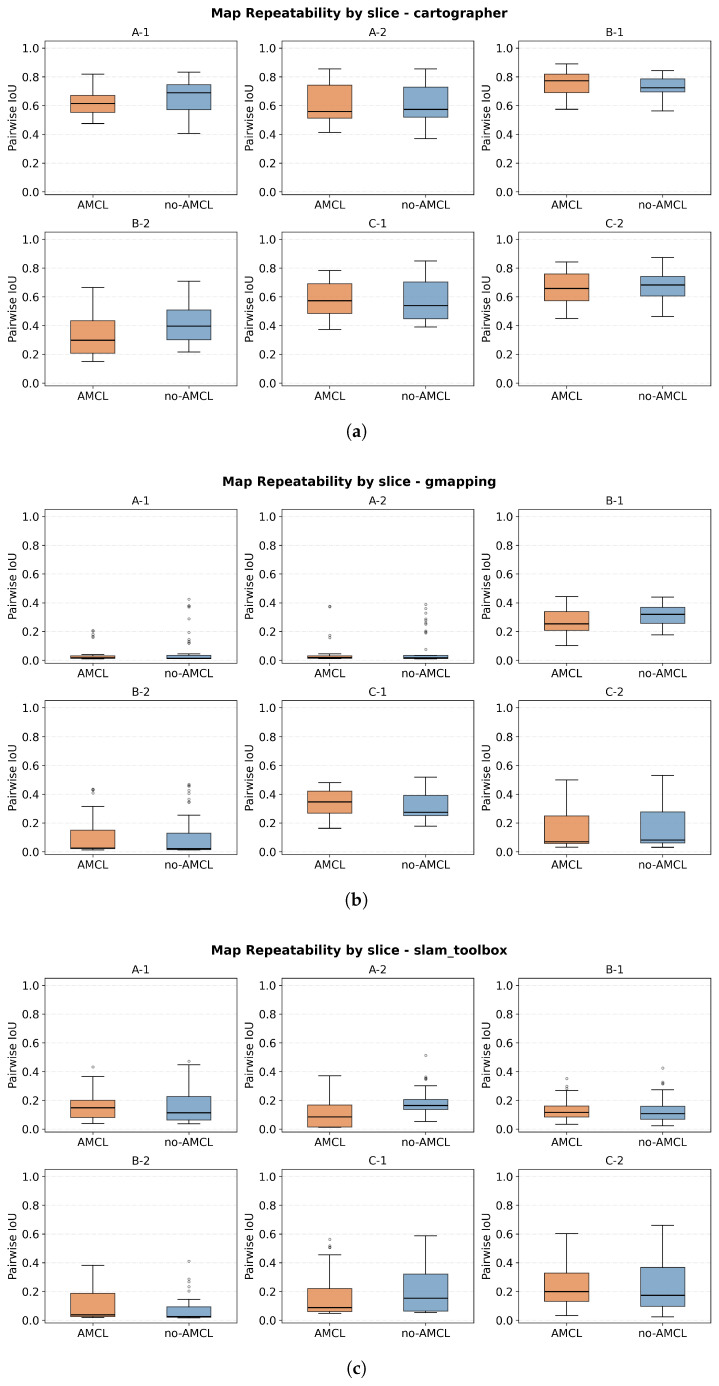
AMCL influence on Mean IoU by measurement slice across environments for quadrupedal Spot and different SLAM backends: (**a**) Cartographer, (**b**) GMapping, (**c**) SLAM Toolbox.

Figure 12 and Figure 13 complement the aggregate view of Table 7 by plotting the mean occupied IoU with 95% confidence intervals as a function of the measurement index (slice) for each algorithm and environment. This temporal decomposition reveals whether AMCL’s effect on map consistency is sustained across all measurement slices or whether it emerges or collapses at specific stages of the traversal.

For the wheeled Panther (Figure 12), Cartographer maintains a consistently higher mean IoU than GMapping and SLAM Toolbox across all environments, with the AMCL-enabled and AMCL-disabled confidence bands separating most clearly in Environment B (Measurement 1), where the tabulated effect size reaches δ=−0.349 and the mean IoU rises from 0.517 to 0.570. In Environment A (Measurement 1), however, the Cartographer AMCL-enabled band lies below the AMCL-disabled band throughout the slice sequence, consistent with the δ=+0.492 degradation reported in Table 7. GMapping and SLAM Toolbox maintain persistently narrow confidence intervals owing to their already-low baseline IoU (0.027–0.194), making any AMCL effect small in absolute terms even when the relative shift is non-negligible.

For the legged Spot (Figure 13), the overall IoU level is higher (Cartographer’s bands frequently exceed 0.6–0.7) and the confidence intervals are wider, reflecting the greater run-to-run variability associated with the legged gait. The most striking feature is the crossover in Environment B (Measurement 2): Cartographer’s AMCL-enabled band dips below the AMCL-disabled band (Table 7: δ=+0.342), while SLAM Toolbox shows the opposite, with slightly improved consistency under AMCL-enabled conditions (δ=−0.262). In Environment A, SLAM Toolbox (Measurement 2) exhibits a marked separation in the later slices, where AMCL progressively degrades structural consistency (reaching a tabulated δ=+0.492), illustrating how AMCL-induced scan jitter accumulates over longer traversals in geometrically repetitive corridors.

#### 3.6.1. Base Algorithm Characteristics

A clear baseline observation across all environments and both platforms is the stark contrast in map consistency between the evaluated SLAM frameworks. Cartographer consistently produced map IoU scores significantly higher (typically ranging from 0.4 to 0.75) than both GMapping and SLAM Toolbox (which often ranged between 0.02 and 0.35). This indicates that Cartographer’s submap-based optimization produces highly repeatable global occupancy grids irrespective of the underlying robot kinematics, whereas the continuous rasterization of GMapping and SLAM Toolbox results in higher structural variance across independent traversals. Notably, the legged Spot platform generally achieved high Cartographer IoU scores, reaching 0.726 under AMCL-disabled conditions and 0.753 under AMCL-enabled conditions in Environment B, Measurement 1.

#### 3.6.2. The Impact of AMCL on Map Consistency

The introduction of AMCL into the localization stack produced mixed, highly environment- and platform-dependent effects on map repeatability.

For the wheeled Panther, AMCL provided a statistically significant improvement in structural consistency primarily in stable environments like Environment B (Measurement 1). Cartographer’s mean IoU increased from 0.517 to 0.570 (δ=−0.349), with similar proportional improvements for GMapping and SLAM Toolbox.

For the legged Spot, the structural effect of AMCL was also selective and backend-dependent. In Environment B, Measurement 1, Cartographer improved from 0.726 to 0.753, whereas GMapping decreased from 0.316 to 0.269. SLAM Toolbox remained nearly unchanged in mean IoU (0.132 to 0.132), although Cliff’s δ suggests a slight distributional shift. This confirms that AMCL does not uniformly improve map consistency even in a structured environment.

Conversely, the use of AMCL can sometimes substantially degrade map consistency. For the Panther in Environment A (Measurement 1), Cartographer experienced a significant structural degradation when AMCL was enabled, with the mean IoU dropping from 0.576 to 0.451. Spot faced similar degradations, most notably with SLAM Toolbox in Environment A (Measurement 2), where the IoU dropped from 0.185 to 0.105 (δ=+0.492).

This phenomenon occurs because the AMCL particle cloud can be drawn into local ambiguities along featureless walls, such as those in Environment A. When this happens, the resulting high-frequency pose corrections cause laser scans to be projected onto the occupancy grid with slight rotational or translational jitter. This jitter may still keep the robot localized along the global path, but it effectively blurs the resulting occupancy grid. As a result, the exact pixel-to-pixel occupied-cell IoU decreases for both wheeled and legged platforms.

## 4. Discussion

### 4.1. Cross-Platform AMCL Behavior Is Conditional, Not Uniform

The results indicate that AMCL does not consistently improve localization repeatability across all scenarios. Its effect is conditioned by environmental topology, platform kinematics, and the internal architecture of the selected SLAM backend. Therefore, AMCL-EKF feedback should be interpreted as a conditional correction source rather than as a universally beneficial drift-reduction mechanism.

Environment B provides the clearest example of a favorable but still platform-dependent response. In this structured and feature-rich environment, AMCL improved selected trajectory results for Spot and improved map consistency for Panther across all three backends in Measurement 1. This suggests that map-referenced corrections are most beneficial when the environment provides sufficiently distinctive geometric features for stable particle-filter localization and scan-to-map association.

Environment A illustrates the opposite behavior. In the long-corridor scenario with repetitive and feature-poor geometry, AMCL often increased trajectory dispersion, particularly for Spot in Measurement 1, where all three SLAM backends degraded under AMCL. This behavior is consistent with perceptual aliasing: the AMCL particle cloud may remain locally plausible while still producing small but persistent pose corrections that are inconsistent with local scan matching. When such corrections are continuously injected into the EKF, they can amplify scan-to-map jitter and reduce both trajectory and map repeatability.

Environment C represents an intermediate but highly heterogeneous case. The mixed corridor-and-room structure does not simply produce moderate AMCL effects; instead, it reveals strong backend- and platform-specific responses. For example, SLAM Toolbox benefits from AMCL on Spot in Measurement 2 but degrades on Panther under the same environment–measurement condition. This indicates that AMCL performance is not determined by the environment alone, but by the interaction between environmental structure, platform motion, and backend-specific use of external odometry.

An important observation from these results is that the final effect of AMCL-EKF fusion is mediated by the selected SLAM backend. The same early pose-correction mechanism can improve, degrade, or leave trajectory repeatability nearly unchanged depending on whether the downstream mapper is Cartographer, GMapping, or SLAM Toolbox. This indicates that the limiting factor is not only the quality of the odometry input or the availability of global map-based corrections, but also how each SLAM backend internally incorporates, filters, and optimizes the supplied pose information.

This backend-dependent response is consistent with the distinct estimation architectures used by the evaluated SLAM systems. Cartographer combines local scan matching with pose-graph optimization, making its performance sensitive to how external pose estimates interact with submap construction and subsequent global optimization. GMapping employs a Rao–Blackwellized particle filter in which odometry affects particle propagation while scan observations determine particle weighting; consequently, externally corrected odometry can alter the balance between motion prediction and observation updates. SLAM Toolbox relies on graph-based scan matching and loop-closure optimization, meaning that externally supplied pose information influences the quality of local constraints that are later incorporated into the global graph. Because each backend integrates motion and observation information differently, the same AMCL-EKF estimate can affect the mapping process in different ways. Therefore, although AMCL-EKF fusion can improve map-referenced pose consistency, its impact on SLAM repeatability depends on how each backend incorporates motion and observation information.

### 4.2. Platform-Specific Interpretation

The most practically relevant cross-platform difference appears in the response to geometrically ambiguous environments. On Panther, AMCL does not provide a uniform improvement; its effect ranges from negligible change in A2/Cartographer (0.326 → 0.333 m), through improvement in A2/GMapping (0.543 → 0.460 m), to degradation in A1/Cartographer (0.294 → 0.662 m) and C2/GMapping (0.441 → 0.812 m). On Spot, the clearest pattern appears in A1, where AMCL increases pairwise ATE for all three backends. This indicates that AMCL should be interpreted as a conditional correction source rather than a platform-specific fail-safe mechanism.

IMU-derived disturbance profiles (Table 3) indicate stronger translational excitation for Spot ($\sqrt{a_{x}^{2}+a_{y}^{2}}$ mean 4.271 vs. 2.804 m/s^2^; P95 10.199 vs. 5.920 m/s^2^), supporting the mechanism that high-frequency body excitation in repetitive geometry can amplify scan-to-map jitter when AMCL corrections are continuously injected.

### 4.3. Trajectory and Map Outcomes Are Coupled but Not Identical

Across slices, trajectory and map effects do not always move together. For example, in Panther/B1, map IoU improves for all three backends, whereas trajectory ATE slightly increases for all three. This confirms that trajectory repeatability and map structural consistency can respond differently to the same AMCL correction strategy.

For example, Spot/B2 shows trajectory degradation for Cartographer and GMapping, and IoU also degrades for Cartographer (0.413→0.336), while remaining nearly unchanged for GMapping (0.103→0.102). In A2 for Spot, SLAM Toolbox shows both trajectory and IoU degradation in map structure (IoU: 0.185→0.105), consistent with AMCL corrections being pulled by local ambiguities.

### 4.4. Computational Costs and Real-Time Applications

All pipelines were executed offline on a workstation to ensure identical processing conditions and to eliminate variability caused by embedded hardware limitations. Consequently, the reported results focus on localization and mapping repeatability rather than on computational performance or execution speed on the robotic platforms.

From a real-time deployment perspective, the evaluated components are widely used in ROS 1 and ROS 2 systems. RF2O and the EKF operate at fixed update rates suitable for continuous online localization, while AMCL provides lower-frequency global pose corrections. The SLAM backends process incoming 2D LiDAR scans incrementally and are commonly deployed in online mapping and localization pipelines.

The additional computational overhead introduced by the AMCL-EKF configuration is primarily associated with particle-filter updates and state-estimation fusion within the EKF. In contrast, the dominant computational burden of the complete pipeline remains in the SLAM backend, particularly in scan matching, submap or graph optimization, and loop-closure detection and correction.

A detailed evaluation of computational cost, processor utilization, memory consumption, and real-time performance on embedded robotic hardware is beyond the scope of this study and is left for future deployment-oriented investigations.

### 4.5. Practical Significance and Deployment Implications

The observed trajectory and map variations have clear practical implications for deployment. Differences on the order of a few centimeters are unlikely to affect coarse inspection mapping, whereas deviations of several decimeters can influence navigation margins in narrow indoor environments, docking accuracy, return-to-base behavior, and the repeatability of localized inspections. More substantial discrepancies, including the meter-level dispersion observed for Spot in Environment A, can alter the reconstructed positions of corridors, doors, and obstacle boundaries, affecting both map interpretation and downstream planning. Similarly, reductions in occupied-cell IoU indicate diminished structural repeatability of walls and obstacles, underscoring the importance of maintaining map consistency when maps are reused for navigation and inspection tasks.

These findings support a platform- and context-aware deployment policy:

- For wheeled systems, AMCL may still be useful as a correction layer, but its activation should be conditioned on online confidence indicators, since the present results show both improvements and degradations depending on the SLAM backend and environment.
- For legged systems in repetitive indoor topology, AMCL should be adaptively weighted or gated to avoid over-correction during high excitation periods.

These findings suggest that a more effective direction is not only to tune existing SLAM backends or improve individual measurements, but to develop an integrated SLAM architecture that explicitly accounts for early global pose corrections. Such an approach should be able to use the potential of AMCL-derived map feedback when the correction is reliable, while suppressing or down-weighting it when the particle filter becomes uncertain, geometrically ambiguous, or inconsistent with local motion estimates. In this sense, the key challenge is to combine the global observability of AMCL with the local smoothness and robustness of odometry-based SLAM without introducing over-corrections.

In practice, AMCL corrections should be conditioned on online reliability indicators (particle-weight concentration, scan-to-map score, innovation magnitude, and EKF covariance growth), rather than applied with fixed weighting.

## 5. Limitations

This study has several limitations that define the scope of the conclusions.

- First, the experiments do not use an external ground-truth trajectory obtained from a motion-capture system, total station, AprilTag-based tracking system, or independently surveyed reference map. Therefore, the reported trajectory metrics should not be interpreted as absolute localization accuracy with respect to the physical world. They quantify repeatability, namely the dispersion between repeated SLAM executions and the consistency of reconstructed occupancy-grid maps. A configuration may therefore be repeatable while still sharing a systematic spatial bias. This limitation is important when interpreting ATE-style values, IoU scores, and the practical meaning of AMCL-induced improvements or degradations.
- Second, the cross-platform comparison is influenced by differences in trajectory execution. Spot followed a predefined autonomous mission replayed using the Boston Dynamics software environment, whereas Panther was human-assisted along the same nominal route. Although path length, velocity profiles, and trajectory-overlap analyses were added to quantify these differences, the two platforms did not execute perfectly synchronized trajectories with identical velocity, turning radius, stopping behavior, or waypoint timing. Consequently, observed differences between Panther and Spot should be interpreted as the combined effect of platform morphology, control mode, locomotion dynamics, and trajectory execution, rather than as a pure wheeled-versus-legged comparison.
- Third, the velocity profiles used in this study are derived from TF pose trajectories rather than from an independent reference velocity measurement. They are useful as common internal descriptors of the executed motion, but they inherit the limitations of the underlying localization estimate, including pose noise, nonuniform update timing, loop-closure corrections, and possible discontinuities introduced by relocalization or graph optimization. They should therefore not be interpreted as ground-truth platform velocities.
- Fourth, the AMCL and EKF parameters were kept fixed to evaluate one practical deployable configuration across all environments, platforms, and SLAM backends. This improves experimental comparability but does not provide a full parameter-sensitivity analysis. In particular, the study does not exhaustively evaluate the influence of particle count, resampling settings, laser-model weights, motion-model noise parameters, AMCL update thresholds, or EKF covariance tuning. Such sensitivity analysis is needed to determine whether the observed degradations can be mitigated by adaptive or environment-specific parameter selection.
- Fifth, computational performance was not evaluated quantitatively on the onboard robotic computer. The experiments were replayed offline on a workstation to provide controlled and repeatable processing conditions. Therefore, CPU utilization, RAM consumption, latency, and real-time factor are not reported as primary metrics. Future deployment-oriented studies should include embedded runtime profiling together with localization and map-repeatability metrics.
- Sixth, the statistical analysis is constrained by the number of independent physical measurements. Although the study contains 720 SLAM executions, many pairwise comparisons are derived from repeated processing of the same raw datasets and therefore are not fully independent physical trials. For this reason, pairwise metrics are interpreted primarily as descriptive distributional measures, while run-level centroid deviations are used where possible for more conservative inference.

Finally, the study is limited to 2D LiDAR occupancy-grid SLAM pipelines in indoor GPS-denied environments. The results should not be generalized directly to full 3D LiDAR-inertial SLAM, visual-inertial SLAM, outdoor rough-terrain navigation, or high-speed locomotion without additional experiments.

## 6. Conclusions

This study presented a repeatability-driven benchmark of AMCL-assisted EKF localization for online 2D LiDAR SLAM using two heterogeneous mobile robots, three SLAM backends, three indoor environments, and 720 SLAM executions. The results show that AMCL-EKF feedback is not universally beneficial. Its effect depends on the environment, platform motion, and the way each SLAM backend incorporates external odometry and pose corrections. Therefore, AMCL should be treated as a conditional correction source rather than an always-on improvement mechanism.

The main conclusions are as follows:

- The SLAM backend has a significant impact on how AMCL-EKF corrections affect final trajectory repeatability. Even though AMCL can improve pose consistency through EKF-based fusion with online SLAM-map-referenced corrections, this improvement does not automatically translate into better repeatability results. The same AMCL-EKF feedback strategy can lead to different outcomes depending on the downstream SLAM backend. Differences in how Cartographer, GMapping, and SLAM Toolbox incorporate external odometry, perform scan matching, handle loop closure, and optimize trajectories can turn the same feedback mechanism into an improvement, a degradation, or a negligible effect.
- AMCL can improve repeatability in regular and geometrically distinctive environments. In Environment B1, Spot shows trajectory improvements for key backends (GMapping: 0.525→0.361 m, δ=−0.213; SLAM Toolbox: 0.210→0.153 m, δ=−0.374). For Panther, map consistency improves for all three algorithms (Cartographer: 0.517→0.570; GMapping: 0.156→0.224; SLAM Toolbox: 0.145→0.190). This indicates that online AMCL feedback can be beneficial when the evolving map provides sufficiently stable and distinctive geometric structure.
- AMCL can also degrade repeatability in ambiguous geometry. In Environment A1 on Spot, all three algorithms show increased trajectory dispersion under AMCL. Selected map configurations also degrade, for example Spot A2/SLAM Toolbox, where occupied cell IoU decreases from 0.185 to 0.105. For Panther, AMCL increases trajectory dispersion in several cases, including A1/Cartographer (0.294 → 0.662 m) and C2/GMapping (0.441 → 0.812 m). These results show that online map feedback can amplify localization and mapping errors when the temporary SLAM map is ambiguous or geometrically repetitive.
- Mobile platform dynamics and execution mode influence the observed SLAM performance. IMU measurements show higher translational excitation for the Spot platform, which is consistent with increased susceptibility to AMCL-induced over-correction in repetitive indoor environments. At the same time, the comparison between Spot and Panther should be interpreted as a combined effect of platform dynamics, locomotion type, and trajectory execution mode, rather than as a purely morphological wheeled-versus-legged comparison.
- The results support confidence-aware AMCL integration rather than fixed always-on fusion. Since AMCL can either improve or degrade repeatability depending on the backend, platform, and environment, future systems should modulate the correction strength online using reliability indicators such as particle-cloud dispersion, scan-to-map likelihood, EKF innovation magnitude, map maturity, motion state, and consistency with local odometry.

Overall, the results show that AMCL-EKF feedback should be treated as a conditional online correction mechanism, not as a universally beneficial localization layer. A promising direction for future work is the development of integrated SLAM architectures that can exploit AMCL-like map feedback when it is reliable, while suppressing or down-weighting it when the online map is immature, ambiguous, or inconsistent with local motion estimates. Future studies should also extend the benchmark to additional 2D and 3D SLAM approaches, higher platform velocities, and more challenging environments such as rough terrain.

## Figures and Tables

**Table 1:** Experimental configurations for Panther and Spot platforms.

Env.	Meas.	Algorithm	Panther		Spot	
			AMCL	No-AMCL	AMCL	No-AMCL
A	1	Cartographer	✓	✓	✓	✓
		GMapping	✓	✓	✓	✓
		SLAM Toolbox	✓	✓	✓	✓
	2	Cartographer	✓	✓	✓	✓
		GMapping	✓	✓	✓	✓
		SLAM Toolbox	✓	✓	✓	✓
B	1	Cartographer	✓	✓	✓	✓
		GMapping	✓	✓	✓	✓
		SLAM Toolbox	✓	✓	✓	✓
	2	Cartographer	✓	✓	✓	✓
		GMapping	✓	✓	✓	✓
		SLAM Toolbox	✓	✓	✓	✓
C	1	Cartographer	✓	✓	✓	✓
		GMapping	✓	✓	✓	✓
		SLAM Toolbox	✓	✓	✓	✓
	2	Cartographer	✓	✓	✓	✓
		GMapping	✓	✓	✓	✓
		SLAM Toolbox	✓	✓	✓	✓

✓ Indicates the configuration was tested with 10 SLAM iterations, totaling 720 SLAM executions.

**Table 2:** Main configuration parameters used for each component of the SLAM pipeline applied.

Component	Configuration Parameters
RF2O	Frequency = 20 Hz.
PointCloud-to-LaserScan	Minimum height = 0.1 m; maximum height = 2.0 m; angle increment inherited from the LiDAR scan; minimum range = 0.45 m; maximum range = 100.0 m.
EKF	Frequency = 20 Hz; two_d_mode = true; fused inputs: RF2O odometry {x, y, z, vx, vy, vyaw}, IMU measurements {x, y, z, roll, pitch, yaw, vroll, vpitch, vyaw} and AMCL pose estimate {x, y}; process noise covariance main diagonal values: [0.05, 0.05, 0.06, 0.03, 0.03, 0.06, 0.025, 0.025, 0.04, 0.01, 0.01, 0.02, 0.01, 0.01, 0.015].
AMCL	Minimum particles = 500; maximum particles = 2000; laser model = likelihood field; laser maximum beams = 60; motion model = DifferentialMotionModel (for Panther), OmniMotionModel (for Spot); update_min_d = 0.25 m; update_min_a = 0.20 rad; resample interval = 1.
Cartographer	Map resolution = 0.05 m; linear update threshold = 0.5 m; angular update threshold = 0.20 rad; default scan-matcher optimization parameters.
GMapping	Map resolution = 0.05 m; linear update threshold = 0.5 m; angular update threshold = 0.20 rad; particles = 30; default scan-matcher optimization parameters.
SLAM Toolbox	Map resolution = 0.05 m; linear update threshold = 0.5 m; angular update threshold = 0.20 rad; scan matching enabled; loop closure enabled with default optimization parameters.

**Table 3:** Raw IMU summary for Environment A, Measurement 1 (computed from attached JSON logs).

Metric	Panther	Spot
Samples [–]	8856	13,136
Duration [s]	206.37	316.50
Sampling rate [Hz]	50.00	50.00
∥ω∥ mean [rad/s]	0.170	0.261
∥ω∥ P95 [rad/s]	0.827	0.621
∥ω∥ max [rad/s]	0.950	2.222
$\sqrt{a_{x}^{2}+a_{y}^{2}}$ mean [m/s^2^]	2.804	4.271
$\sqrt{a_{x}^{2}+a_{y}^{2}}$ P95 [m/s^2^]	5.920	10.199
$\left|∥a∥-9.81\right|$ mean [m/s^2^]	1.311	2.293
$\left|∥a∥-9.81\right|$ P95 [m/s^2^]	3.433	8.753
Dominant freq. in $a_{z}$ [Hz]	5.05	8.60

**Table 4:** Mean linear and angular velocity statistics comparison between platforms.

Env.	Meas.	Panther (Wheeled)				Spot (Legged)			
		Linear		Angular		Linear		Angular	
		Mean (m/s)	Std. Dev.	Mean (rad/s)	Std. Dev.	Mean (m/s)	Std. Dev.	Mean (rad/s)	Std. Dev.
A	1	1.09	0.01	0.073	0.003	0.90	0.01	0.069	0.003
A	2	0.37	0.01	0.033	0.001	0.91	0.00	0.067	0.002
B	1	1.02	0.01	0.040	0.002	0.92	0.01	0.048	0.001
B	2	0.38	0.01	0.017	0.001	0.45	0.00	0.028	0.001
C	1	0.94	0.02	0.064	0.003	0.92	0.00	0.072	0.002
C	2	0.37	0.01	0.028	0.002	0.90	0.01	0.072	0.002

**Table 5:** Trajectory scale: path length statistics comparison between platforms.

Env.	Meas.	Panther (Wheeled)		Spot (Legged)	
		Mean (m)	Std. Dev. (m)	Mean (m)	Std. Dev. (m)
A	1	229.35	4.39	277.00	4.45
A	2	216.84	8.11	271.59	3.04
B	1	225.55	3.64	219.32	2.73
B	2	220.41	6.08	286.77	1.94
C	1	181.74	3.36	163.34	2.11
C	2	168.15	6.52	168.33	2.11

**Table 6:** Mean pairwise ATE (m), Cliff’s δ, and statistical-test *p*-values comparison between platforms.

Env.	Meas.	Algorithm	Panther (Wheeled)					Spot (Legged)				
			No-AMCL	AMCL	Cliff’s δ	Welch *p*	MW *p*	No-AMCL	AMCL	Cliff’s δ	Welch *p*	MW *p*
A	1	Cartographer	0.294	0.662	+0.645	7.72 × 10^−8^	0.038	1.867	2.196	+0.114	0.336	0.140
		GMapping	0.962	0.809	−0.223	0.285	0.473	2.448	2.794	+0.265	0.388	0.241
		SLAM Toolbox	0.198	0.330	−0.135	0.041	1.000	2.250	2.260	+0.043	0.979	0.571
	2	Cartographer	0.326	0.333	+0.028	0.804	0.571	0.683	0.736	−0.052	0.661	0.273
		GMapping	0.543	0.460	−0.234	0.015	0.186	0.596	0.515	−0.206	0.132	0.186
		SLAM Toolbox	0.230	0.282	+0.142	0.041	0.910	0.425	0.388	−0.032	0.547	0.678
B	1	Cartographer	0.278	0.288	+0.102	0.790	0.427	0.168	0.182	−0.003	0.536	0.734
		GMapping	0.573	0.636	+0.225	0.482	1.000	0.525	0.361	−0.213	0.002	0.910
		SLAM Toolbox	0.192	0.216	+0.105	0.235	0.623	0.210	0.153	−0.374	0.003	0.054
	2	Cartographer	0.236	0.360	+0.132	0.006	0.345	0.374	0.541	+0.481	2.79 × 10^−5^	0.031
		GMapping	0.531	0.372	−0.358	0.002	0.089	0.339	0.397	+0.147	0.092	0.571
		SLAM Toolbox	0.240	0.226	+0.007	0.485	0.791	0.170	0.195	+0.108	0.154	0.850
C	1	Cartographer	0.421	0.419	+0.052	0.971	0.623	0.315	0.321	+0.000	0.881	0.791
		GMapping	0.472	0.462	−0.024	0.827	0.473	0.455	0.402	−0.160	0.174	0.521
		SLAM Toolbox	0.210	0.200	+0.001	0.646	0.850	0.411	0.424	+0.078	0.811	0.571
	2	Cartographer	0.381	0.338	+0.061	0.434	0.385	0.459	0.434	−0.036	0.638	0.970
		GMapping	0.441	0.812	+0.662	7.52 × 10^−9^	0.076	0.444	0.490	+0.168	0.295	0.162
		SLAM Toolbox	0.217	0.333	+0.418	9.94 × 10^−5^	0.307	0.281	0.220	−0.294	0.128	0.064

Note: Mean ATE values, Cliff’s *δ*, andWelch *p* are computed from pairwise run-to-run ATE distributions. Because pairwise samples are not statistically independent, these values are interpreted as descriptive distributional comparisons. The Mann–Whitney *p* value is computed from run-level centroid deviations.

**Table 7:** Mean pairwise Occupied Map IoU, effect size (Cliff’s δ), and statistical-test *p*-values comparison between platforms.

Env.	Meas.	Algorithm	Panther (Wheeled)					Spot (Legged)				
			No-AMCL	AMCL	Cliff’s δ	Welch *p*	MW *p*	No-AMCL	AMCL	Cliff’s δ	Welch *p*	MW *p*
A	1	Cartographer	0.576	0.451	+0.492	1.67 × 10^−5^	5.85 × 10^−5^	0.657	0.625	+0.196	0.133	0.110
		GMapping	0.027	0.026	+0.062	0.826	0.611	0.070	0.037	+0.029	0.087	0.815
		SLAM Toolbox	0.194	0.177	+0.117	0.518	0.341	0.161	0.154	−0.004	0.774	0.974
	2	Cartographer	0.575	0.582	−0.032	0.733	0.796	0.609	0.610	+0.018	0.977	0.885
		GMapping	0.056	0.061	−0.155	0.610	0.205	0.074	0.043	−0.102	0.132	0.406
		SLAM Toolbox	0.061	0.066	−0.064	0.483	0.600	0.185	0.105	+0.492	9.81 × 10^−5^	5.85 × 10^−5^
B	1	Cartographer	0.517	0.570	−0.349	0.010	0.004	0.726	0.753	−0.196	0.126	0.110
		GMapping	0.156	0.224	−0.288	0.004	0.018	0.316	0.269	+0.309	0.010	0.012
		SLAM Toolbox	0.145	0.190	−0.253	0.038	0.039	0.132	0.132	−0.059	0.974	0.634
	2	Cartographer	0.697	0.641	+0.207	0.012	0.090	0.413	0.336	+0.342	0.012	0.005
		GMapping	0.261	0.202	+0.336	0.002	0.006	0.103	0.102	−0.245	0.983	0.045
		SLAM Toolbox	0.100	0.090	+0.108	0.304	0.379	0.068	0.112	−0.262	0.040	0.032
C	1	Cartographer	0.621	0.561	+0.141	0.088	0.249	0.575	0.585	−0.063	0.721	0.611
		GMapping	0.159	0.153	+0.103	0.762	0.401	0.319	0.336	−0.127	0.404	0.302
		SLAM Toolbox	0.240	0.294	−0.251	0.136	0.040	0.207	0.171	+0.129	0.275	0.294
	2	Cartographer	0.493	0.528	−0.153	0.261	0.211	0.678	0.656	+0.126	0.311	0.305
		GMapping	0.158	0.157	+0.001	0.928	0.994	0.172	0.138	+0.173	0.221	0.158
		SLAM Toolbox	0.105	0.080	+0.207	0.033	0.090	0.243	0.233	−0.045	0.781	0.717

Note: Mean IoU, Cliff’s *δ*, Welch *p*, and Mann–Whitney *p* are computed from pairwise occupied-cell IoU distributions. Since each map contributes to multiple pairwise comparisons, the statistical-test values are interpreted as descriptive distributional comparisons. Negative Cliff’s *δ* indicates higher IoU under AMCL-enabled conditions, whereas positive values indicate lower IoU under AMCL-enabled conditions.

**Figure A1:**
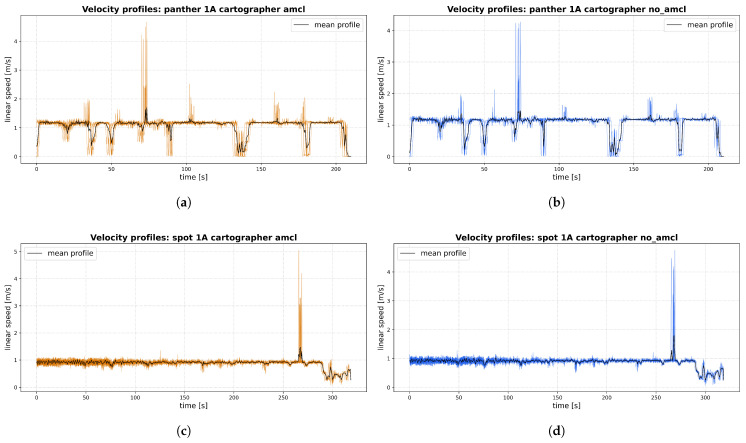
Linear velocity profiles for Cartographer runs with calculated mean values (black) for Environment A, Measurement 1: (**a**) Panther, AMCL-enabled; (**b**) Panther, AMCL-disabled; (**c**) Spot, AMCL-enabled; (**d**) Spot, AMCL-disabled.

**Figure A2:**
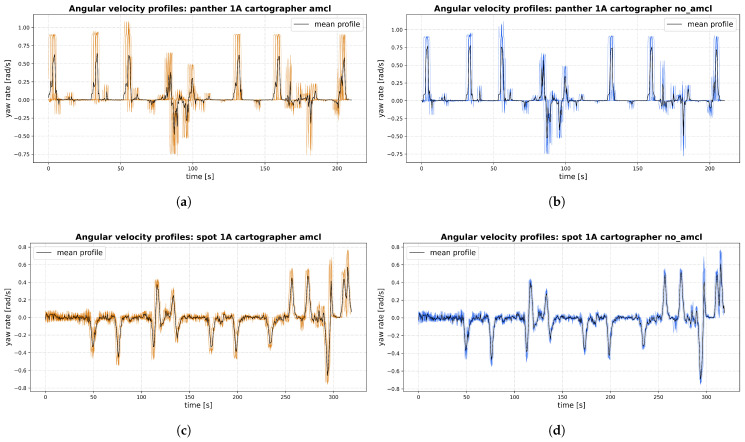
Angular velocity profiles for Cartographer runs with calculated mean values (black) for Environment A, Measurement 1: (**a**) Panther, AMCL-enabled; (**b**) Panther, AMCL-disabled; (**c**) Spot, AMCL-enabled; (**d**) Spot, AMCL-disabled.

**Figure A3:**
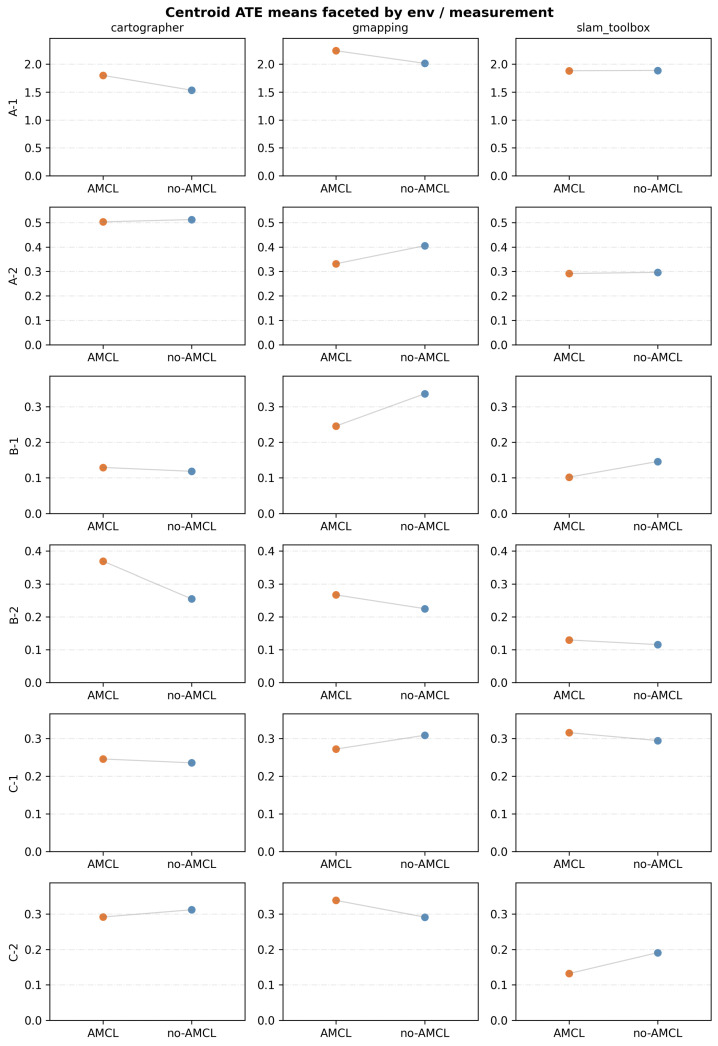
Mean centroid ATE-style dispersion for the Spot platform across all environments, measurements, and SLAM backends. Each small panel corresponds to one environment–measurement condition; each point is the mean run-level deviation from the condition mean trajectory. Lower values indicate greater trajectory repeatability.

**Figure A4:**
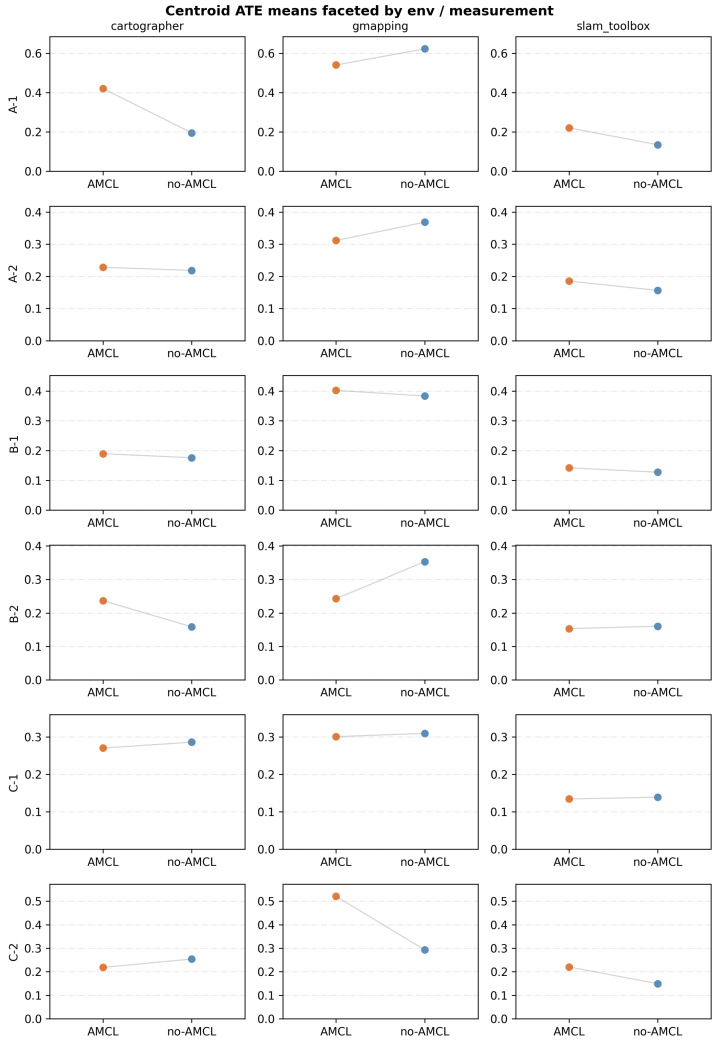
Mean centroid ATE-style dispersion for the Husarion Panther platform across all environments, measurements, and SLAM backends. Each small panel corresponds to one environment–measurement condition; each point is the mean run-level deviation from the condition mean trajectory. Lower values indicate greater trajectory repeatability.

**Figure A5:**
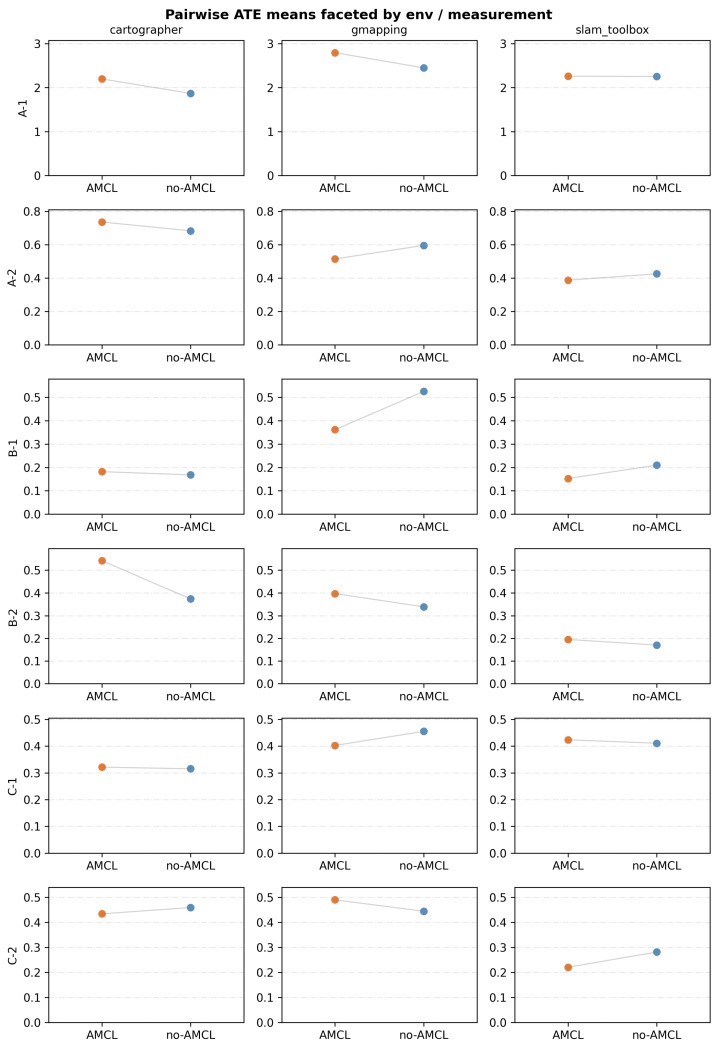
Mean pairwise ATE-style dispersion for the Spot platform across all environments, measurements, and SLAM backends. Each small panel corresponds to one environment–measurement condition; each point summarizes all unordered run-pair comparisons within that condition. Lower values indicate greater trajectory repeatability.

**Figure A6:**
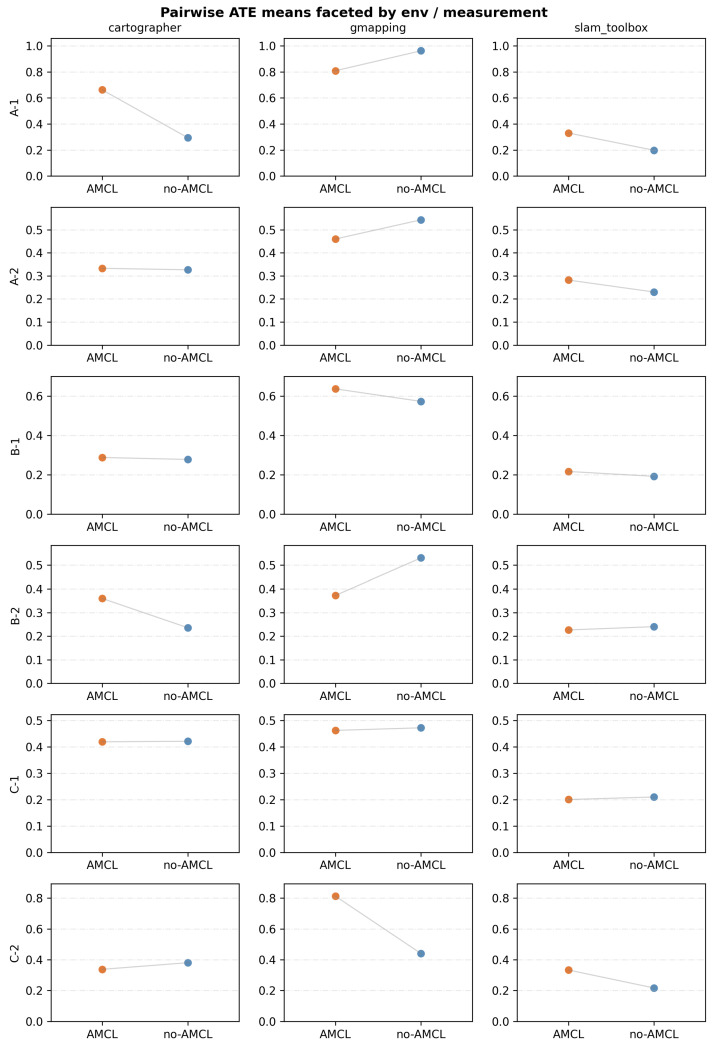
Mean pairwise ATE-style dispersion for the Panther platform across all environments, measurements, and SLAM backends. Each small panel corresponds to one environment–measurement condition; each point summarizes all unordered run-pair comparisons within that condition. Lower values indicate greater trajectory repeatability.

**Figure A7:**
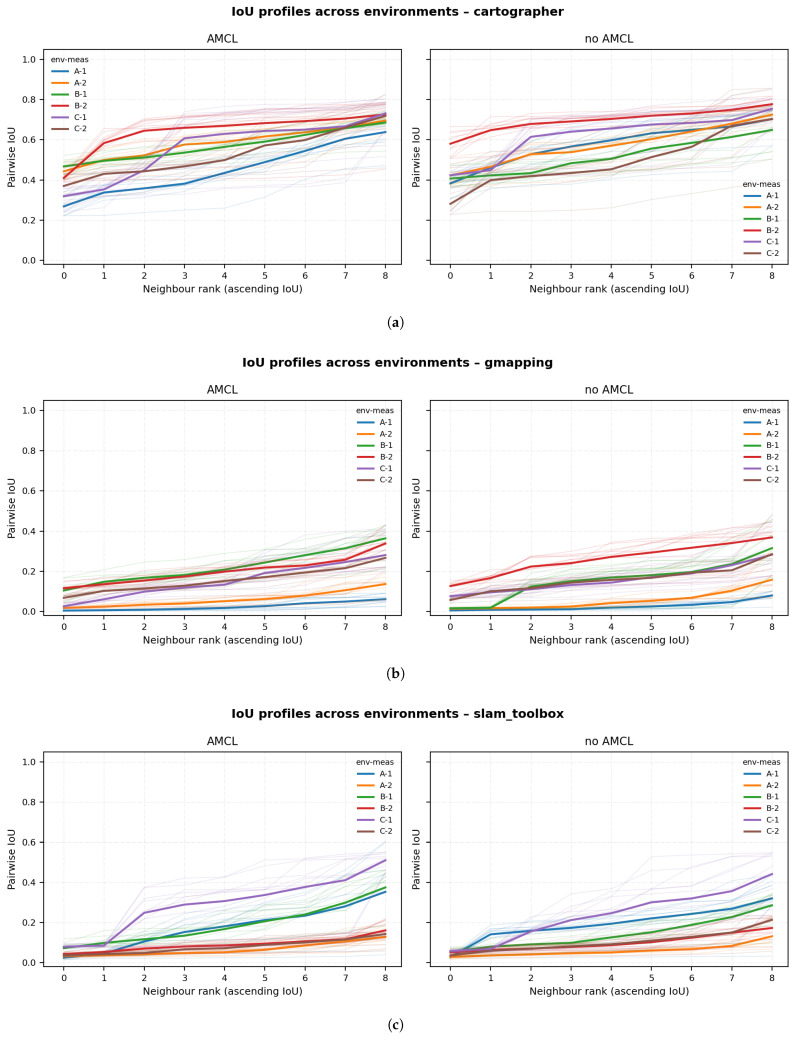
Pairwise occupied-cell IoU profiles for Panther across the evaluated environment-measurement conditions: (**a**) Cartographer, (**b**) GMapping, and (**c**) SLAM Toolbox. The left and right columns show AMCL-enabled and AMCL-disabled conditions, respectively. Colored curves correspond to environment–measurement subsets, and the horizontal axis orders pairwise IoU values within each subset. Higher IoU indicates greater structural map consistency.

**Figure A8:**
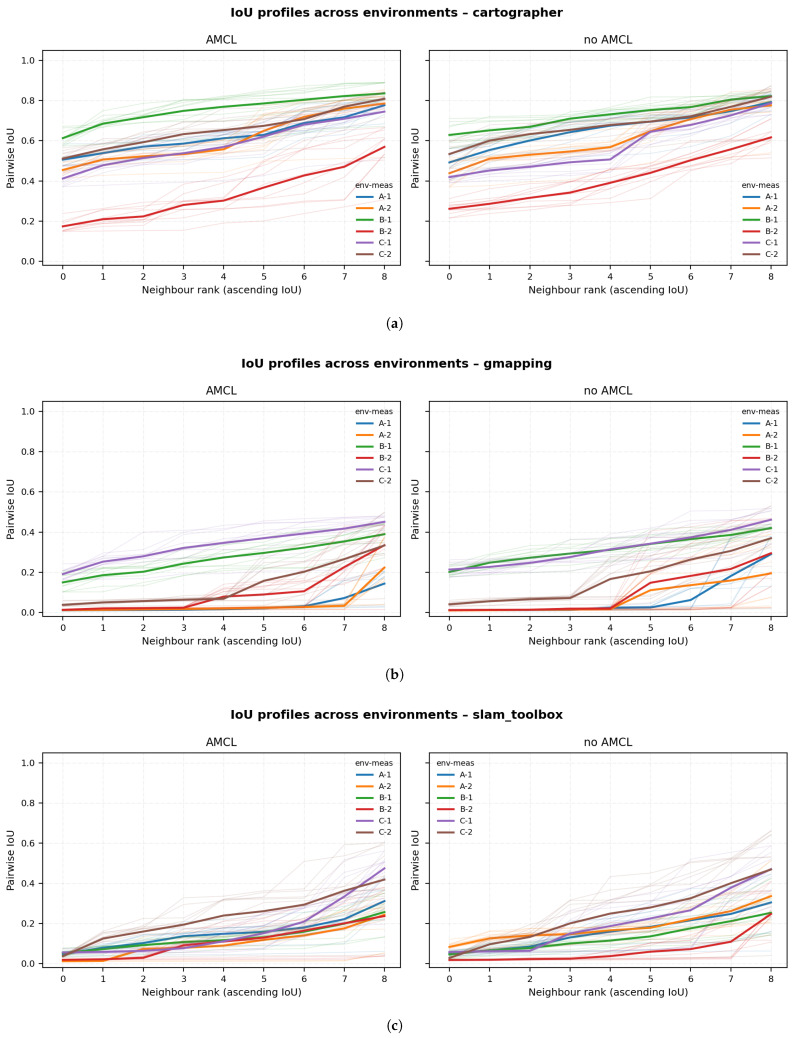
Pairwise occupied-cell IoU profiles for Spot across the evaluated environment–measurement conditions: (**a**) Cartographer, (**b**) GMapping, and (**c**) SLAM Toolbox. The left and right columns show AMCL-enabled and AMCL-disabled conditions, respectively. Colored curves correspond to environment–measurement subsets, and the horizontal axis orders pairwise IoU values within each subset. Higher IoU indicates greater structural map consistency.

**Figure A9:**
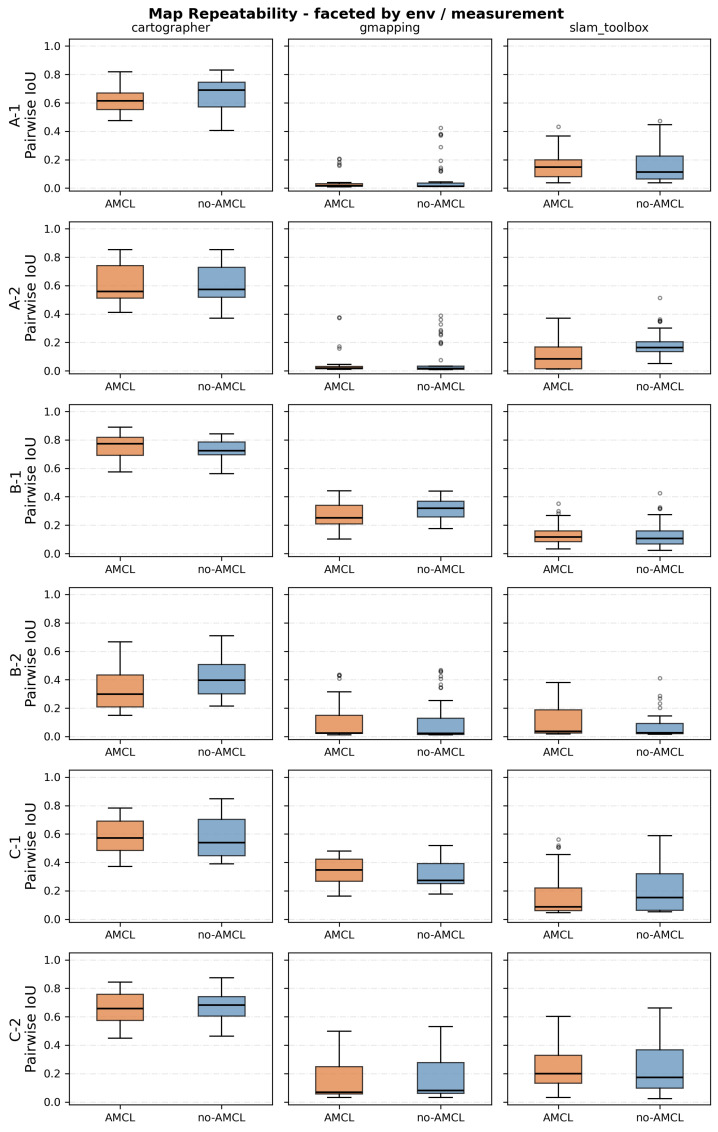
Faceted occupied-cell IoU distributions for the Boston Dynamics Spot platform across all environments, measurements, and SLAM backends. Each small panel corresponds to one environment–measurement-backend condition and compares pairwise map-repeatability values for AMCL-enabled and AMCL-disabled runs. Higher IoU indicates greater structural map consistency.

**Figure A10:**
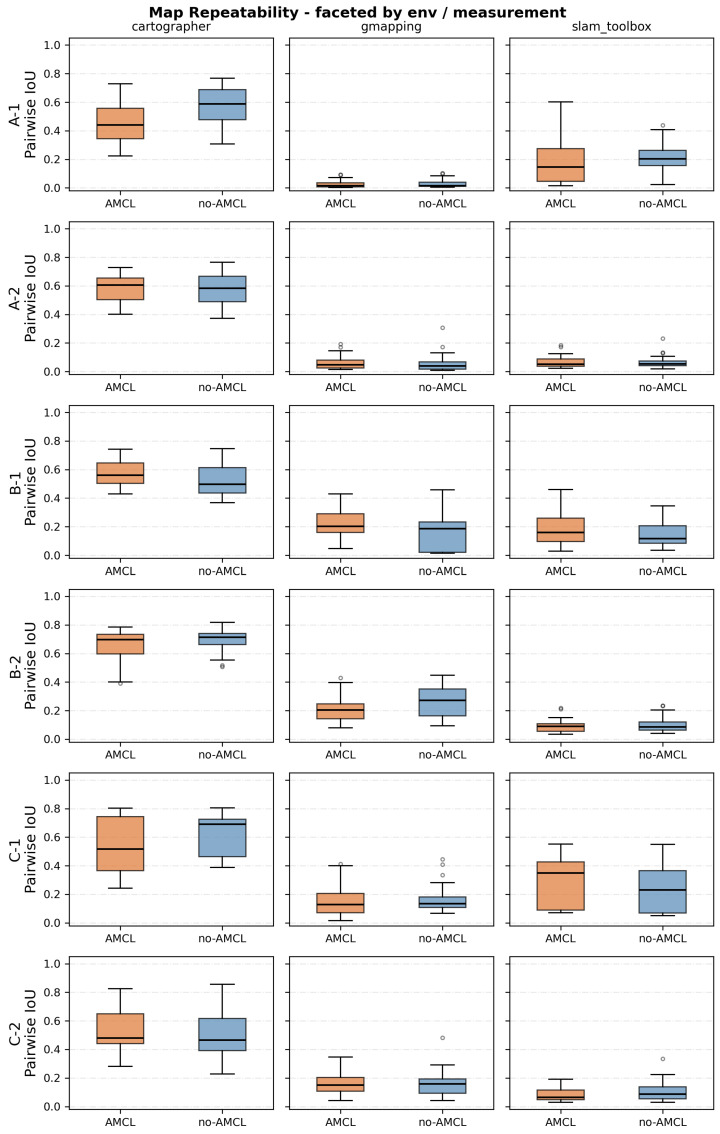
Faceted occupied-cell IoU distributions for the Husarion Panther platform across all environments, measurements, and SLAM backends. Each small panel corresponds to one environment–measurement–backend condition and compares pairwise map-repeatability values for AMCL-enabled and AMCL-disabled runs. Higher IoU indicates greater structural map consistency.

**Figure A11:**
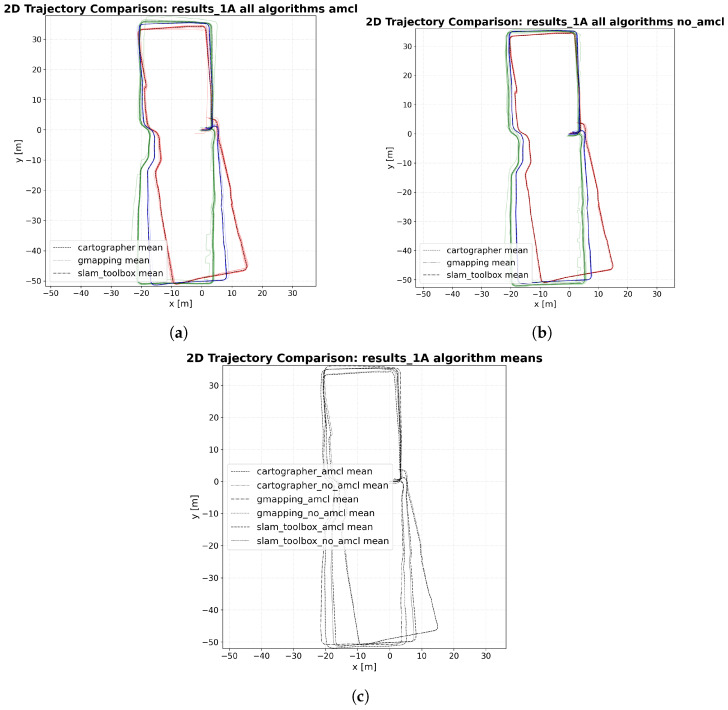
Trajectories per run with mean for all algorithms (Panther, A-1): (**a**) AMCL-enabled, (**b**) AMCL-disabled, (**c**) mean values per algorithm. Red, Blue and Green colors in (**a**,**b**) correspond to Cartographer, Gmapping and SLAM Toolbox algorithms.

## Data Availability

The original data presented in the study are openly available at https://doi.org/10.58032/AGH/FKFLPY (RODBUK Dataverse repository). Available data consists of raw measurements (ROS 2 bag), processed results (csv and png files) and SLAM results (txt and json logs, maps etc) which are organized hierarchically by platform, environment, and iteration. Due to huge size of the raw data, ROS 2 bag files are not included in the repository but are available on request from the corresponding author.

## Abbreviations

The following abbreviations are used in this manuscript:

AGH	AGH University of Krakow
AMCL	Adaptive Monte Carlo Localization
ATE	Absolute Trajectory Error
CI	Confidence Interval
DoF	Degrees of Freedom
EKF	Extended Kalman Filter
GPS	Global Positioning System
ICP	Iterative Closest Point
IMU	Inertial Measurement Unit
IoU	Intersection over Union
LiDAR	Light Detection and Ranging
PGM	Portable Gray Map (image format)
RMSE	Root Mean Square Error
ROS	Robot Operating System
RPE	Relative Pose Error
SE(2)	Special Euclidean Group in 2D
SLAM	Simultaneous Localization and Mapping
SO(2)	Special Orthogonal Group in 2D
SSIM	Structural Similarity Index Measure
SVD	Singular Value Decomposition
TF	Transform (ROS coordinate frame)

## Appendix A

### Appendix A.1. Velocity Profile Metrics

This section presents a representative velocity profile example for one environment–measurement–algorithm combination. Since each run provides two profiles (linear and angular), the complete analysis contains 720×2=1440 velocity profiles in total; the complete set is available in the published dataset. The example shown here was selected to illustrate that, even within the same environment and algorithm, the temporal structure of motion can differ markedly between platforms and localization modes.

Environment A, Measurement 1, and the Cartographer algorithm were chosen as the representative case. The linear velocity profiles are shown in Figure A1 and the angular velocity profiles in Figure A2. Each subplot shows the individual repetitions together with the group mean profile.

### Appendix A.2. Trajectory Repeatability Metrics

This section presents detailed trajectory-repeatability visualizations for all platform-backend combinations, including absolute trajectory error (ATE), root mean square error (RMSE), and maximum deviation, under AMCL-enabled and AMCL-disabled conditions. These metrics are reported for each environment and measurement slice, enabling a comprehensive analysis of AMCL’s effect on localization performance under varying conditions.

#### Appendix A.2.1. Mean Centroid ATE

Figure A3 shows the mean centroid ATE for the Spot platform across all environments, measurement iterations, and SLAM backends, under AMCL-enabled and AMCL-disabled conditions. Figure A4 shows the corresponding results for Husarion Panther. Each facet corresponds to one algorithm–environment–measurement combination. Lower values indicate higher trajectory repeatability relative to the mean trajectory. Together, these visualizations enable a comprehensive comparison of AMCL’s impact on trajectory consistency across conditions, highlighting platform-, environment-, and backend-dependent differences.

#### Appendix A.2.2. Mean Pairwise ATE

Figure A5 shows the mean pairwise ATE for the Spot platform across all environments, measurement iterations, and SLAM backends, under AMCL-enabled and AMCL-disabled conditions. Figure A6 shows the corresponding results for Husarion Panther. Each facet corresponds to one algorithm–environment–measurement combination. Lower values indicate higher trajectory repeatability relative to other runs within the same condition. Together, these visualizations enable a comprehensive comparison of AMCL’s impact on trajectory consistency across conditions, highlighting platform-, environment-, and backend-dependent differences.

### Appendix A.3. Map Repeatability Metrics

#### Appendix A.3.1. Intersection over Union (IoU) Profiles

Figure A7 and Figure A8 show the IoU profiles across environments under AMCL-enabled and AMCL-disabled conditions for the Panther and Spot platforms, respectively, and different SLAM backends. Each profile illustrates how AMCL influences map consistency across varying conditions, highlighting differences in performance between the two platforms and SLAM approaches.

#### Appendix A.3.2. Faceted Mean IoU with Confidence Intervals

Figure A9 and Figure A10 show the faceted mean IoU with confidence intervals for the Panther and Spot platforms, respectively, across all environments, measurement iterations, and SLAM backends, under AMCL-enabled and AMCL-disabled conditions. Each facet corresponds to one algorithm–environment–measurement combination. This visualization allows for a comprehensive comparison of AMCL’s impact on map consistency across different conditions, highlighting differences in performance between the two platforms and SLAM approaches.

### Appendix A.4. Trajectory and Map Plots per Run

Due to the large volume of data and the public availability of the dataset, we have chosen to feature only representative plots from a single measurement, rather than including all generated trajectories and maps (720 unique runs with collected data).

Figure A11 shows the trajectories for all runs of Panther in Environment A1 across all SLAM backends, under AMCL-enabled and AMCL-disabled conditions, and the mean trajectory per algorithm. This visualization allows for a direct comparison of trajectory consistency across runs and conditions, highlighting the influence of AMCL on trajectory repeatability in this specific environment. Each subplot corresponds to a different condition: (a) AMCL-enabled, (b) AMCL-disabled, and (c) mean trajectories per algorithm, providing insights into the overall performance trends across the different SLAM approaches and the effect of AMCL on trajectory consistency.

Figure A12 shows the maps generated for Spot in all environments (a single run), generated by the SLAM Toolbox algorithm under AMCL-enabled conditions.
